# Transcriptomic and Genetic Analyses Identify the Krüppel-Like Factor Dar1 as a New Regulator of Tube-Shaped Long Tendon Development

**DOI:** 10.3389/fcell.2021.747563

**Published:** 2021-12-16

**Authors:** Quentin Laurichesse, Blandine Moucaud, Lilia Laddada, Yoan Renaud, Krzysztof Jagla, Cédric Soler

**Affiliations:** GReD Institut - UMR CNRS 6293 / INSERM U1103 University of Clermont-Auvergne, Clermont-Ferrand, France

**Keywords:** tubulogenesis, leg disc, tendon, myogenesis, krüppel-like factor, dar1, cytoskeleton, drosophila

## Abstract

To ensure locomotion and body stability, the active role of muscle contractions relies on a stereotyped muscle pattern set in place during development. This muscle patterning requires a precise assembly of the muscle fibers with the skeleton via a specialized connective tissue, the tendon. Like in vertebrate limbs, *Drosophila* leg muscles make connections with specific long tendons that extend through different segments. During the leg disc development, cell precursors of long tendons rearrange and collectively migrate to form a tube-shaped structure. A specific developmental program underlies this unique feature of tendon-like cells in the *Drosophila* model. We provide for the first time a transcriptomic profile of leg tendon precursors through fluorescence-based cell sorting. From promising candidates, we identified the Krüppel-like factor Dar1 as a critical actor of leg tendon development. Specifically expressed in the leg tendon precursors, loss of *dar1* disrupts actin-rich filopodia formation and tendon elongation. Our findings show that Dar1 acts downstream of Stripe and is required to set up the correct number of tendon progenitors.

## Introduction

The musculoskeletal system comprises numerous cellular components including muscles and tendons. The assembly of these components is tightly controlled to achieve a stereotyped functional architecture. Acquisition of muscle shape and pattern thus directly relies on where the muscle fibers are anchored to the skeleton via specialized structures, the tendons. Tendons are required not only to transmit the muscle contraction force to the skeleton but also to set up a functional musculoskeletal system. The importance of coordinated development of tendons and more generally of connective tissues (CT) and muscles into an integrated system is well documented in different model organisms (Kardon et al., 2003; Schweitzer et al., 2010; Hasson, 2011; Kardon, 2011; Nassari et al., 2017; Felsenthal et al., 2018). Research on different models indicates that final muscle patterning relies on several extrinsic elements (Schnorrer and Dickson, 2004; Hasson, 2011; Valdivia et al., 2017; Laurichesse and Soler, 2020). In *Drosophila* embryo, inducing ectopic muscle attachment sites interferes with myotube guidance (Vorbrüggen and Jäckle, 1997) and several secreted or membrane-associated proteins have been described as mediating muscle migration toward the tendon cells (Callahan et al., 1996; Prokop et al., 1998; Kramer et al., 2001; Swan et al., 2004; Schnorrer et al., 2007; Ordan et al., 2015). In avian models, transplantation studies showed that myoblast patterning was dependent on the surrounding connective tissues with Tcf-4-expressing CT cells establishing the muscle prepattern (Kardon et al., 2002, 2003; Rinon et al., 2007; Mathew et al., 2011). These observations support the hypothesis that the correct patterning of musculo-skeletal system depends not only on the features of specific muscles but also on specific CT features. Yet, in contrast to muscles, the genetic program that controls the formation of morphologically and functionally distinct tendons has not been investigated.

Like vertebrates, *Drosophila* shows a variety of morphologically distinct tendons. Larval monofiber muscles are linked to the exoskeleton (Volk and VijayRaghavan, 1994; Frommer et al., 1996) through a single cell attachment site, whereas large clusters of tendon cells specified in the wing disc epithelium anchor the massive flight muscles in the adult thorax (Fernandes et al., 1996). In the fly leg, the appendicular movements are ensured by the connection between muscles and long internal tendons (Miller, 1950; Soler et al., 2004). In *Drosophila*, Stripe (Sr)/Egr-like is the key transcription factor of tendon differentiation and a hallmark of all tendon cells (Volk and VijayRaghavan, 1994; Fernandes et al., 1996; Frommer et al., 1996; Vorbrüggen and Jäckle, 1997; Ghazi et al., 2003; Soler et al., 2004). Although a few other transcription factors are specifically expressed in tendon sub-populations such as *apterous* in wing disc-associated tendons or *GCM/Glide* in embryos, their contribution to the acquisition of characteristic properties of specific tendons remains elusive.

**FIGURE 1:**
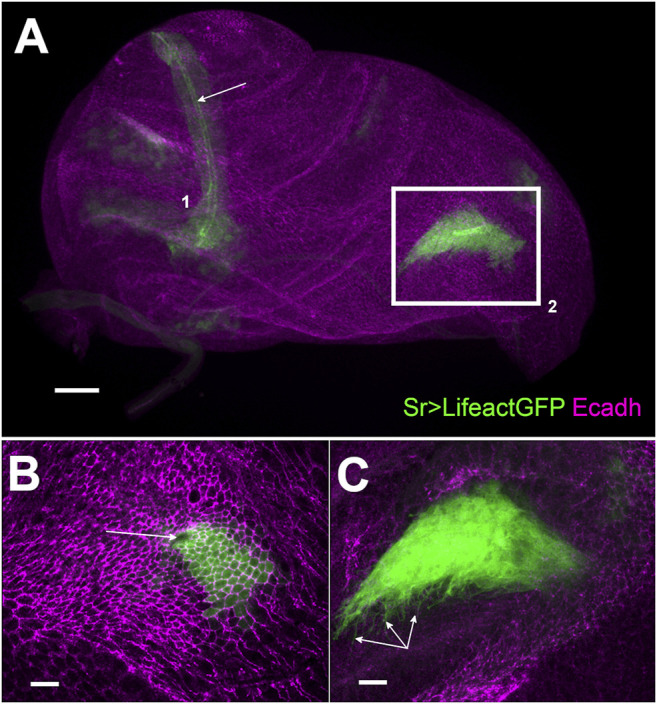
Development of long tendons from 0 h APF leg disc visualized using sr-gal4 driven Lifeact.GFP expression **(A)** z-projection of selected optical sections of 0 h APF leg disc. Long tendons form from clusters of epithelial cells (stained with E-cadherin in magenta) expressing sr-gal4>UAS-Lifeact.GFP (green), long tendon (lt) of the tarsi (1) has elongated from the joint between tarsus T5 and pretarsus. Apical accumulation of Lifeact.GFP outlines the tube lumen (arrow). In the dorsal femur (box 2), the tibia levator long tendon (tilt) has started to invaginate and elongate. Scale bar 30 μm **(B)** and **(C)** High magnification of selected confocal planes (from bo×2). Scale bar 10 μm **(B)** Surface view: we can observe apical constriction of Lifeact.GFP cells, revealed by E-cadherin staining, around the invaginating pit (arrow) forming the tube lumen **(C)** deeper *Z* planes reveal dense arborization of actin-based filopodia at the basal side of the invaginating tendon (arrows).

Amongst the various tendons observed in Drosophila, the leg tendons show morphological and developmental similarities with the long tendons of the autopod in the mouse. They extend from the most distal part of the leg and elongate through the leg segments, where they are associated with extrinsic muscles (Huang, 2017; Huang et al., 2015; Soler et al., 2004; Watson et al., 2009). Strikingly, the morphogenesis of these leg long tendons in *Drosophila* shares many similarities with the tubulogenesis that occurs during tracheal or salivary gland formation (Girdler and Röper, 2014; Hayashi and Kondo, 2018; Maruyama and Andrew, 2012). First, the tendon cells undergo apical constriction followed by invagination without migration (Laddada et al., 2019). The cells then collectively migrate with the extension of basal protrusions, leading to the elongation of a tube-shaped tendon (Figure 1). It has also been suggested that like for the developing salivary glands (Vining et al., 2005), elongating tendons could interact with surrounding tissues, especially with the myoblasts (Soler et al., 2016, 2004). We have already shown that long tendons originate from tendon precursors that are selected among the cells of the leg segmental joints expressing *odd-skipped* (Laddada et al., 2019). While Odd is sufficient to induce the primary invagination of the epithelial cells (Hao et al., 2003; Ibeas and Bray, 2003), Stripe is required to make these cells competent to migrate and form a long tube-shaped tendon (Laddada et al., 2019).

To gain a better understanding of the developmental mechanisms of appendicular tendon formation, we undertook a transcriptomic analysis of the long tendon precursors. RNA sequencing of these cells at the onset of long tendon formation enabled us to identify the associated expression of tube morphogenesis-related genes, the downregulation of which recapitulated phenotypes observed in other models of tubulogenesis. This finding functionally validates *Drosophila* leg tendon development as a new model to study tube morphogenesis. We subsequently performed a genetic screen to identify key transcription factors involved in the developmental program of these specific tendons. Among the positive candidates, we identified *dar1* (*dendritic arbor reduction 1*), a gene coding for a DNA-binding protein belonging to the Krüppel-like transcription factor (KLF) family. In *Drosophila*, Dar1 had previously been shown to determine the multipolar morphology of post-mitotic neurons (Ye et al., 2011; Wang et al., 2015) and to downregulate the proliferation of intestinal stem cells (Wu et al., 2018). Here, we show that *dar1* is not expressed in the tendon precursors of the flight and larval muscles but that its expression is restricted to leg tendon precursors. Our functional analysis revealed that long tendon development was severely impaired after *dar1* knockdown. The number of cytoplasmic protrusions was drastically reduced, and the elongation, but not the invagination of tendon cell precursors was compromised. Importantly, our results show that in the absence of Dar1, the initial expression of *stripe* (*sr*) in subsets of leg disc epithelial cells is not compromised but that the final number of Stripe-positive tendon cells is strongly reduced. In view of its critical effect on tendon development, we describe the role of Dar1 as a key component of tube-shaped tendon morphogenesis, which is also required to set up the correct number of leg tendon cells.

## Results

### Transcriptomic Profiling of Appendicular Long Tendon Precursors Reveals a Gene Expression Signature of Tubulogenesis

We had previously shown that precursors of the long tendons are clusters of epithelial cells selected among the cells of leg segmental joints. The Notch pathway initiates *stripe* expression, the key factor in tendon cell differentiation, in the leg tendon precursors (Soler et al., 2004; Laddada et al., 2019).

These Sr-positive cells invaginate and migrate to form the tube-shaped long tendon between the end of the larval stages and the first hours of pupal formation. To gain a better understanding of the molecular mechanisms underlying these dramatic cell morphological changes, we undertook a whole transcriptome RNA-seq analysis of these cells at the onset of pupation (0 h APF). Total RNAs were prepared from FACS-isolated sr-gal4>UAS-GFP cells from 0 h APF leg discs and processed for whole-transcriptome RNA-seq (GSE169313), followed by bioinformatic analysis (for cell-sorting and RNAseq detailed protocols and validations see Materials and Methods and Supplementary Material). With the FPKM cut-off at 10 for reliable detection of gene expression, we found that a wide array of genes 5,479) were significantly expressed in sr-gal4>UAS-GFP cells, probably reflecting the developmental plasticity exhibited by *Drosophila* imaginal disc cells (McClure and Schubiger, 2007). However, gene ontology (GO) analysis of these genes showed an over-representation of the GO terms related to “locomotion” and “muscle structure development” with numerous genes known to be involved in muscle attachment site development and/or the formation of myotendinous junctions (Table 1). Considering GOs related to pathways, we found an over-representation of Notch, Wnt and MAPK pathways, consistent with our previous results and those of others, showing that these pathways play a key role in tendon development in *Drosophila* (Yarnitzky et al., 1997; Soler et al., 2004; Lahaye et al., 2012; Laddada et al., 2019). Finally, this analysis also pointed to GO terms relative to tube morphogenesis such as “epithelial tube morphogenesis” or “regulation of tube size”, supporting our earlier assertion that long tendon development shared common features with salivary gland or tracheal tube morphogenesis (Girdler and Röper, 2014; Hayashi and Kondo, 2018; Laddada et al., 2019). In order to functionally validate this point, we crossed the sr-gal4 line with UAS-RNAi or UAS-Dominant Negative lines directed against genes known to regulate many aspects of cell adhesion, migration or maintenance of tissue integrity during morphogenetic processes such as tubulogenesis (Supplementary Table S1). Expression of most of these RNAi or dominant negative transgenes strongly impacted the normal development at larval or pupal stages and RNAi against genes such as the *β*PS integrin totally disrupted the long tendon formation (data not shown). Interestingly, knocking down the expression of genes controlling the length of developing tubes (Luschnig et al., 2006; Kerman et al., 2008) such as *serpentine*, *vermiform* or *lolal*, led to an excessive elongation of the long tendons in the mature leg (Supplementary Figure S1). Altogether, these results validate our approach to identifying new regulators of appendicular long tendons and support a pivotal role of tubulogenesis in their development.

**TABLE 1:** List of over-represented GO from RNAseq analysis of sr-gal4>UAS-GFP cells. 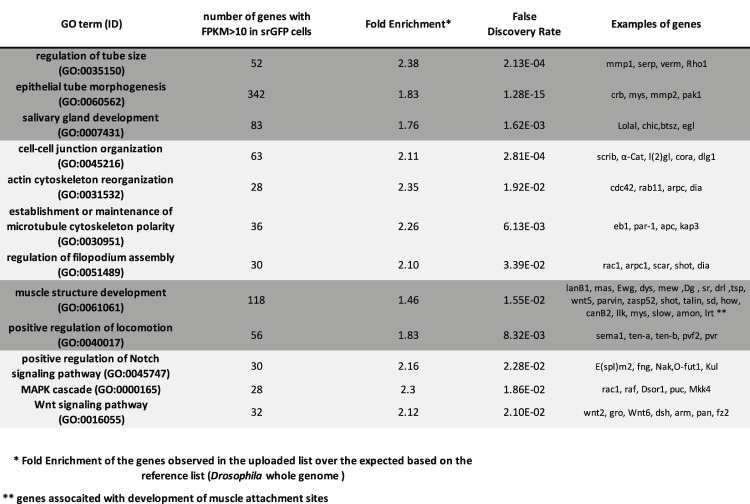

### Deciphering the Core Genetic Program of Leg Tendon Development

Because transcription factors are fundamental in controlling the developmental program to build any structure during development, we carried out a lethality and climbing-based *in vivo* RNAi screen targeting the genes that encode for TFs that we found specifically enriched in our RNAseq data. Remarkably, of the 5,479 genes with FPKM>10, transcripts of 31 genes encoding for predicted transcription factors (Rhee et al., 2014) exhibited more than 1.5-fold higher levels of expression in the GFP + cells than in the whole leg disc cells (input), strongly suggesting that these TFs have a relevant specific role in the development of the leg long tendon (Supplementary Table S2). We performed this RNAi screen by crossing tendon-specific UAS-Dicer2; sr-gal4 line with UAS-RNAi lines targeting these 31 candidates from two independent libraries: TRiP (Bloomington Stock Center) and VDRC (Vienna Stock Center), when available. To limit the number of false positives due to off-target effects, we did not include UAS-line RNAi from the VDRC stock center, for which more than two off-targets were predicted (this was the case for three genes). In this way, we crossed 53 UAS-RNAi lines to target the 31 selected genes, and we could test two different lines for 71% of them (22/31) (Supplementary Table S2). F1 generation were screened for developmental lethality and/or climbing defect (for the detailed screen protocol, see Materials and Methods). Out of the 31 candidates analyzed, seven showed a significant embryonic (or early larval stage) lethality, with more than 20% of embryos unable to hatch or dying before their third larval stage, 12 displayed a pupal lethality score higher than 20%, and 14 showed a climbing defect. Thus, nearly 65% (20/31, including *stripe* itself) of the candidate genes were positive for at least one readout and for eight of them this result was obtained with two different RNAi lines (Table 2). Within these positive hits, we found *odd* and *drm* both members of the *odd-skipped* gene family that act downstream of the Notch pathway to promote leg tendon growth (Laddada et al., 2019). We also identified *esg*, which interferes with the Notch pathway during stem cell maintenance/differentiation (Korzelius et al., 2014), and which is required during tracheal morphogenesis (Miao and Hayashi, 2016). Moreover, it has been shown that *esg* misexpression in embryonic epidermal cells (including muscle attachment sites) can interfere with the development of muscle attachment sites (Staudt et al., 2005). These positive genes strengthen our strategy to identify new regulatory factors of leg long tendon development. Other candidates, including six uncharacterized predicted transcription factors (CG numbers), have never been reported or investigated in tendon development.

**TABLE 2:** List of positive candidate genes based on RNAi screen. 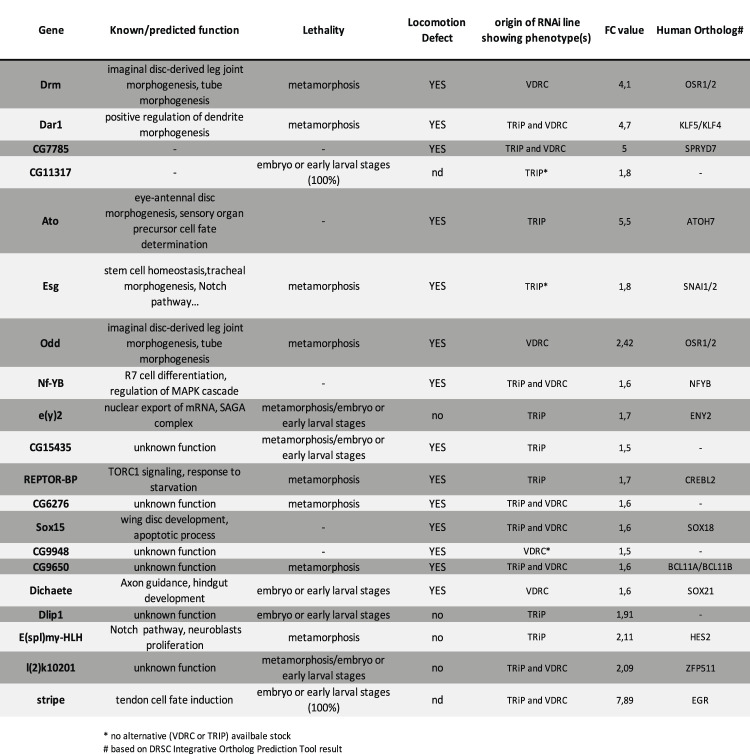

We then considered the vertebrate orthologs of these newly-highlighted TFs in order to identify functional evolutionary conservation. We note that of the 14 conserved genes, the orthologs of *dar1* (KLF5 and KLF4) and *CG9650* (BCL11 A) were shown to be differentially regulated in limb tendon cells during mouse development in two independent studies (Havis et al., 2014; Liu et al., 2015). Strikingly, for each of these genes, the expression of two different RNAi lines gave significant adult climbing defect and/or metamorphosis but no embryonic lethality (Table 2). These observations strongly suggest that these two genes are likely candidates for the specific development of appendicular long tendons. Below we analyze the expression and function of *dar1* characterized by high transcript enrichment in our RNAseq datasets.

### *dar1* Is Expressed in Leg Tendons but Not in Embryonic and Flight Muscle Attachment Sites

**FIGURE. 2:**
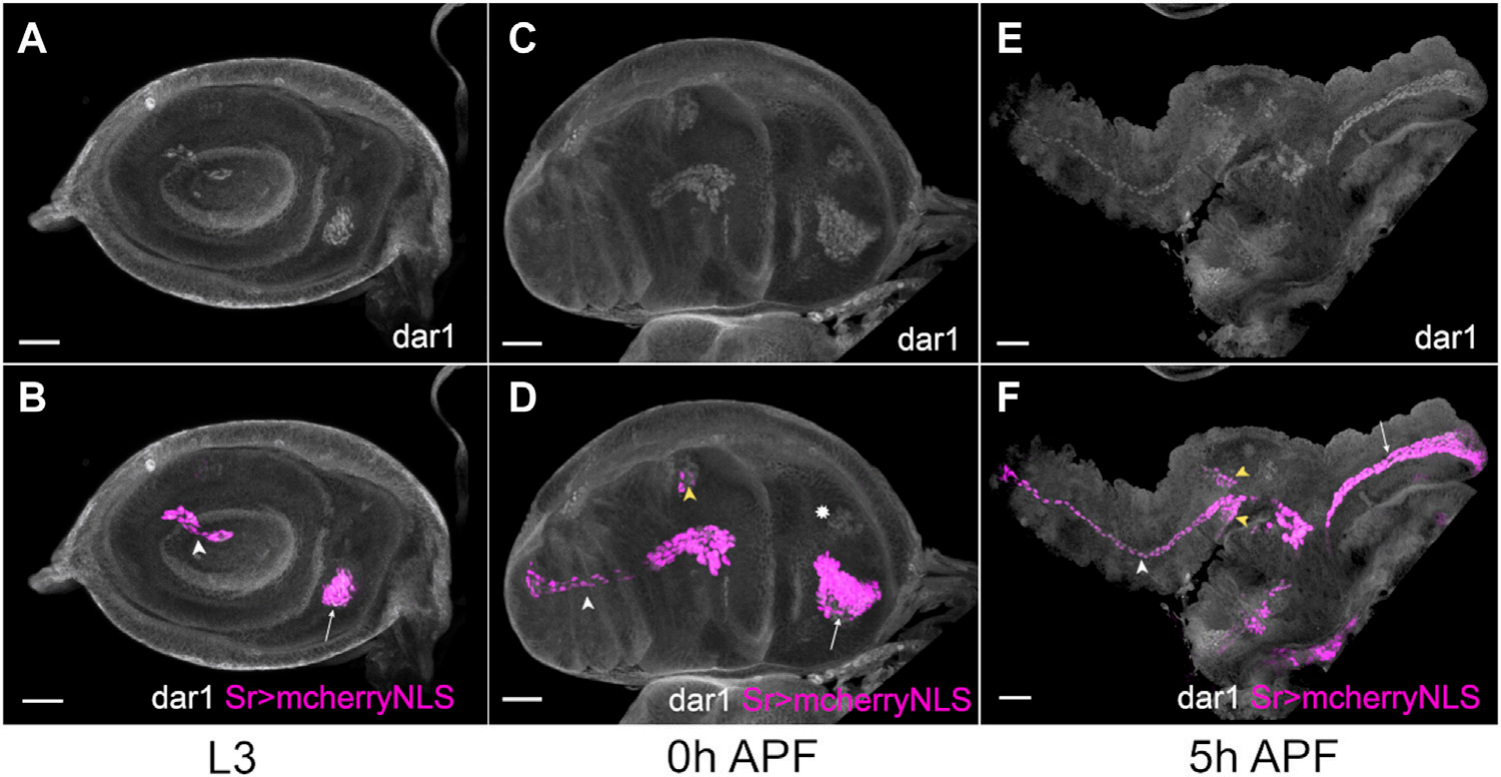
*dar1* expression pattern co-localizes with *stripe* expression in *Drosophila* leg disc. Selected optical sections of sr-gal4>UAS-mcherryNLS (magenta) leg discs immunostained with anti-Dar1 (gray) at different steps of development **(A,B)** In L3 leg disc, *dar1* and *mcherryNLS* expressions colocalize in cells prefiguring the future lt in the tarsi (white arrowhead) and the future tilt in the dorsal femur (arrow). This co-expression is maintained at 0 h APF **(C,D)** and 5 h APF **(E,F)** in elongating tendons, whereas newly-specified sr > mcherry-positive cells, in tibia segment notably, also start to express *dar1* (yellow arrowheads). Note that *dar1* is also expressed at a low level in the apparent chordotonal organ at 0 h APF (asterisk). Scale bar 30 μm.

To confirm the expression of *dar1* in the leg disc tendon, we performed immunostaining on sr-gal4>UASmcherryNLS leg discs from third larval instar to 5 h after pupal formation (APF) using Dar1 antibody (Ye et al., 2011). *dar1* expression colocalizes with *stripe* expression domains, within the cells corresponding to the specified long tendons of the leg (Figure 2). *dar1* was first found in the two earliest clusters of Sr-positive cells that would form the long tendon of the tarsi (lt) and in tibia levator tendon (tilt) in the dorsal femur (Figures 2A,B). Subsequently, during early pupation this expression was maintained in lt and tilt and new Sr-positive clusters corresponding to other long tendons in different leg segments started to be specified (Figures 2C–F). Importantly, *dar1* expression was observed in all these clusters and correlated with our RNA-sequencing data generated from tendon precursors at early pupal stage (0,2 h APF), which showed a high specific enrichment of *dar1* expression in sr-gal4>GFP cells compared to IP (FC = 4.7), strengthening the reliability of our data. To determine whether *dar1* was a hallmark of all tendon cells in *Drosophila,* we also analyzed its expression in embryos and in wing discs (Supplementary Figure S2). In embryos, we could not detect Dar1 in sr-gal4>UASmcherryNLS positive tendon cells (Supplementary Figure S2A,B). Likewise, tendon precursors of flight muscles in the wing disc did not show any *dar1* expression (Supplementary Figure. S2C,D).

All in all, these results strongly suggest that Dar1 plays a specific role in the establishment of these unique long tendons in *Drosophila*.

### Correct Adult Muscle Patterning Required *dar1* Tendon Expression

**FIGURE 3:**
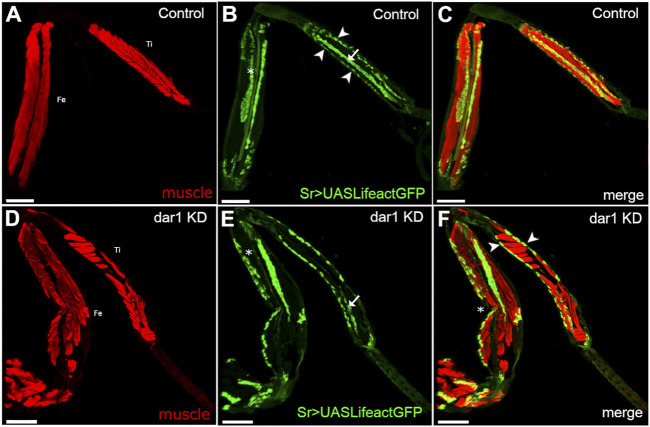
Knockdown of *dar1* expression in long tendons alters myotendinous architecture of adult leg. Combination of confocal images from adult leg cryosections (Tibia: Ti, Femur: Fe); muscles are stained with stained phalloidin (red). Tendons are visualized with Lifeact.GFP (green) **(A–C)** In each segment of control sr-gal4,dar1^3010/+^>UAS-Lifeact.GFP legs, major muscles display a feather-like pattern. For instance, fibers of the muscle in the ventral tibia are attached on one side to the tarsi depressor long tendon (arrow in B) and on the other to an individual cuticle muscle attachment site (arrowheads in B) **(D–F)** on this cryosection of sr-gal4, *dar1* ^3010/+^>UAS-Lifeact.GFP,UAS-dar1RNAi leg, the tilt in the dorsal femur is absent (asterisk in E compared to B) and the long ventral tendon appears shorter in the tibia (arrow in B and E). Alteration or absence of these long tendons leads to the misattachments of muscle fibers with both ends attached to cuticular attachment sites (arrowheads in F) leading to the formation of transversal fibers. Note that disruption of myotendinous architecture seems to affect the morphology of the leg, with a pinch in the femur segment (asterisk in F). number of leg segment observed after cryosection (control *n* = 5, dar1 KD *n* = 6). Scale bar 100 μm.

To determine whether the adult locomotion defects observed in tendon-specific *dar1* KD could be explained by an abnormal development of the appendicular musculotendinous system, we analyzed adult leg myotendinous architecture in dar1 KD flies. In sr-gal4>UAS-Lifeact.GFP, UAS-dar1RNAi flies, we observed heterogenous results with only a few tendons partially affected per fly (data not shown). This observation is likely due to a residual expression of the targeted gene, which is expected when using RNA interference. We therefore decided to use the sr-gal4>UAS-Lifeact.GFP, UAS-dar1RNAi flies associated with a heterozygous null dar1^3010^ allele to potentialize dar1RNAi efficiency and facilitate the subsequent analysis. As shown by longitudinal cryosections along the proximo-distal axis of the sr-gal4,dar1^3010/+^>UAS-Lifeact.GFP, UAS dar1RNAi legs, long tendons in all segments were often shortened or even missing, and so muscle pattern was strongly affected (Figure 3). In control legs, major muscles labeled by phalloidin, displayed a feather-like pattern with each fiber attached on one side to a long tendon and on the other one to an individual cuticle muscle attachment site (cMAS). By contrast, when long internal tendons were affected, both ends of a muscle fiber were anchored to a cMAS, generating a transverse fiber. This is clearly visible in the ventral tibia where the long tendon appears much shorter in the visualized cryosections (Figure 3E). Thus, in the proximal region of this segment, both ends of the muscle fibers are attached to cMAS, whereas in the distal part, the fibers still connect to the shortened remaining ventral long tendon (Figure 3F). We could also observe a highly disorganized muscle pattern in the dorsal femur that coincided with no visible tilt in this cryosection. Our observations thus indicate that when *dar1* expression is specifically lowered in leg tendon precursors, resulting long tendons are severely affected, leading to abnormal muscle patterning. Moreover, even in the absence of long tendons, the muscle fibers can still attach to cuticle via cMAS, suggesting that the latter, like those of flight and embryonic muscles, do not required Dar1 activity.

### Dar1 Is Required for Tubulogenesis-like Morphogenesis of Leg Tendons

**FIGURE. 4:**
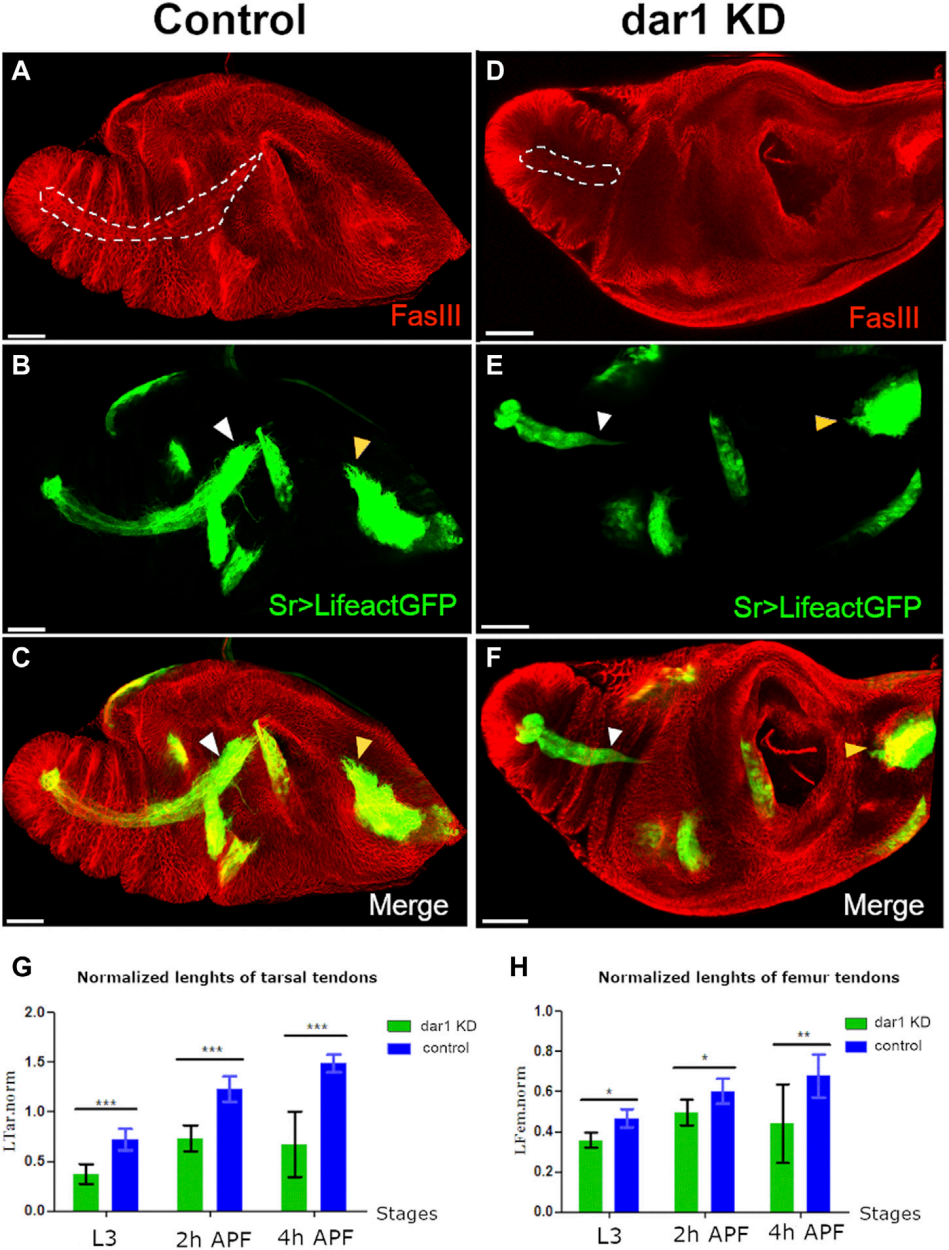
Dar1 is required for the elongation of long tendons. Confocal images of sr-gal4,dar1^3010/+^>UAS-Lifeact.GFP **(A–C)** and sr-gal4,dar1^3010/+^>UAS-Lifeact.GFP,UAS-dar1RNAi **(D–F)** leg discs at 3 h APF immunostained with anti-fasIII. On this optical section, the long elongating tendon of the tarsi is revealed by UAS-Lifeact.GFP and FasIII (dashed line A and D). In *dar1* KD leg disc **(D–F)**, the tarsal lt appears much shorter than in the control (white arrowheads in B,C and E,F). At this stage of development, other tendons are less elongated than the one in the tarsi and therefore their difference in length between control and *dar1* KD is not as obvious, except for the tilt in the dorsal femur (yellow arrowhead in B,C and E,F), which also appears shorter **(G,H)** Graphs showing the lengths of the lt in tarsi (G) and tilt femur (H) in control leg discs versus and *dar1* KD leg disc at three time points: late L3 (control *n* = 11, *dar1* KD *n* = 11), 2 h APF (control *n* = 10, *dar1* KD *n =* 17) and 4hAPF (control *n =* 5, *dar1* KD *n =* 5). Error bars represent s.d.; *p < 0.05, **p < 0.01 and ***p < 0.001 (Bonferroni test). Scale bar 40 μm.

**FIGURE 5:**
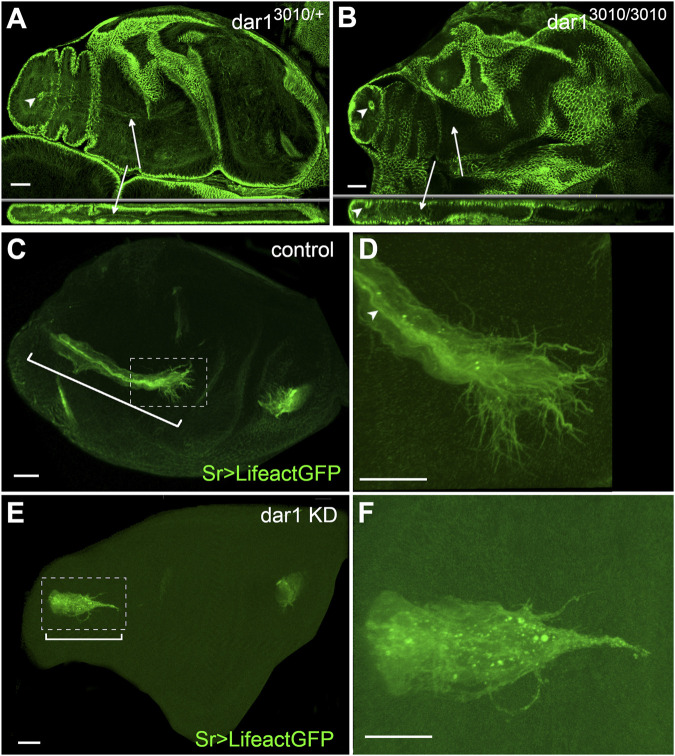
Dar1 is not required for the epithelium invagination but is needed for actin-rich filopodia arborization of long tendons **(A,B)** Confocal sections of 2 h APF leg discs immunostained with anti-dlg **(A)** selected optical sections of control *dar1* ^*3010/+*^ heterozygous leg disc. dlg accumulation reveals lumen aperture on the most distal tarsal segment (arrowhead) and also stained a long internal structure corresponding to tarsal lt (arrow) **(B)** In all observed leg discs from rare *dar1* ^*3010/3010*^ homozygous mutant escapers, local constriction of the epithelium still underlines the local invagination of the epithelial cells, but no elongating internal structure is formed (arrows) (number of samples, control *dar1* ^*3010/+*^ *n* = 10, *dar* ^3010/3010^ *n* = 5) **(C–F)** Confocal sections of 0 h APF leg discs from sr-gal4,dar1^3010/+^>UAS-Lifeact.GFP and sr-gal4,dar1^3010/+^>UAS-Lifeact.GFP,UAS-dar1RNAi 2 h APF pupae. On these selected optical sections, in *dar1* KD condition tarsal lt (bracket in E) appears much shorter than in the control (bracket in C) **(D)** and **(F)** are higher magnifications from **(C)** and **(E)** respectively **(D)** In the control leg disc, leading cells at the distal part of the elongating tendon display numerous actin-rich protrusions at their basal membrane and apical accumulation of actin underlines the tube lumen (arrowhead) **(F)** In the *dar1* KD leg disc, filopodia arborization is systematically affected with a marked decrease in protrusion number (number of samples, control *n* = 10, *dar1* KD *n* = 17). Overall Lifeact.GFP distribution is strongly affected and tube lumen cannot be distinguished in *dar1* KD. Scale bar 20 μm.

To better characterize the role of Dar1 in long tendon development, we focused our analysis on the time when tendon precursors started to express *dar1*, from the L3 larval stage to the early hours of pupation. We specifically analyzed the tarsal lt, extending from pre-tarsus to femur, and the tilt in the dorsal femur. These two tendons form earliest: they are specified at the early third larval instar and then invaginate and elongate until the first hours of metamorphosis (Laddada et al., 2019; Soler et al., 2004). Tendon specific depletion of *dar1* led to apparent shortening of these elongated structures in early metamorphosis (Figures 4A–F). To quantify this phenotype, we measured the relative size of the lt and tilt (for measurement details see Materials and Methods) at three developmental time points (late L3, 2 and 4 h APF) in control (sr-gal4,dar1^3010/+>^UASLifeact.GFP) and *dar1* KD (sr-gal4,dar1^3010^/+>UAS-Lifeact.GFP, UASdar1RNAi) leg discs. For each of these developing tendons, we observed a significant reduction of their length at the three different time points when *dar1* expression was lowered (Figures 4G,H). The tarsal tendon was very significantly shortened as early as late L3, whereas the difference in size of the tilt in dorsal femur was more pronounced at later stages (4 h APF). This is probably because tarsal lt is specified and starts to elongate slightly earlier than the one in the dorsal femur (Soler et al., 2004) and is therefore longer at a given time point. Thus, the length difference compared with control was greater in earlier stages for the tarsal lt than for the tilt in dorsal femur. We infer that the apparent smaller size of the tendons after *dar1* KD becomes more and more significant as the tendons elongate, supporting a potential function of Dar1 in this process. Likewise, 2 h APF leg discs from homozygote *dar1* ^*3010*^ rare escapers (less than 1% of the larva reach the pupal stage and none of them survive for more than a few hours APF), stained against discs large (dlg) septate junction marker, showed no visible elongating structure (Figures 5A,B). Remarkably, in the location of the most distal tarsal segment where lt normally starts to develop, we could still see an accumulation of dlg protein prefiguring the local folding of the epithelium and the lumen formation (Figures 5A,B). These observations indicate that Dar1 is not needed for initial steps of epithelium invagination but is then required for the tube-like tendon elongation.

Besides this elongation defect, *dar1* RNAi expression in long tendon precursors results in a severe depletion of filopodia as revealed by UAS-Lifeact.GFP expression (Figures 5C–F). For instance, the total number of filopodia at the tip of the tarsal lt in control 2 h APF disc was systematically greater than 30 (*n* = 10), whereas in *dar1* KD leg discs, tarsal lt systematically displayed fewer than five remaining protrusions (*n =* 17). This result indicates that the reduction of *dar1* expression directly or indirectly affects the formation of actin-rich filopodia. Moreover, while the tendon lumen in control leg discs is underlined by apical Lifeact.GFP accumulation, in *dar1* KD leg disc, we observed a fluorescent dotty pattern reflecting a general actin disorganization or mislocalization.

Taken together, our results show that Dar1 participates in the cellular events required for actin cytoskeleton organization and that dar1 KD reduces the number of cytoplasmic protrusions.

### Dar1 Is Required to Set up the Correct Number of Sr-Positive Cells

#### dar1 Depletion Induces Loss of Sr-Positive Progenitors Without Affecting Number of Mitotic Cells nor Inducing Apoptosis

**FIGURE 6:**
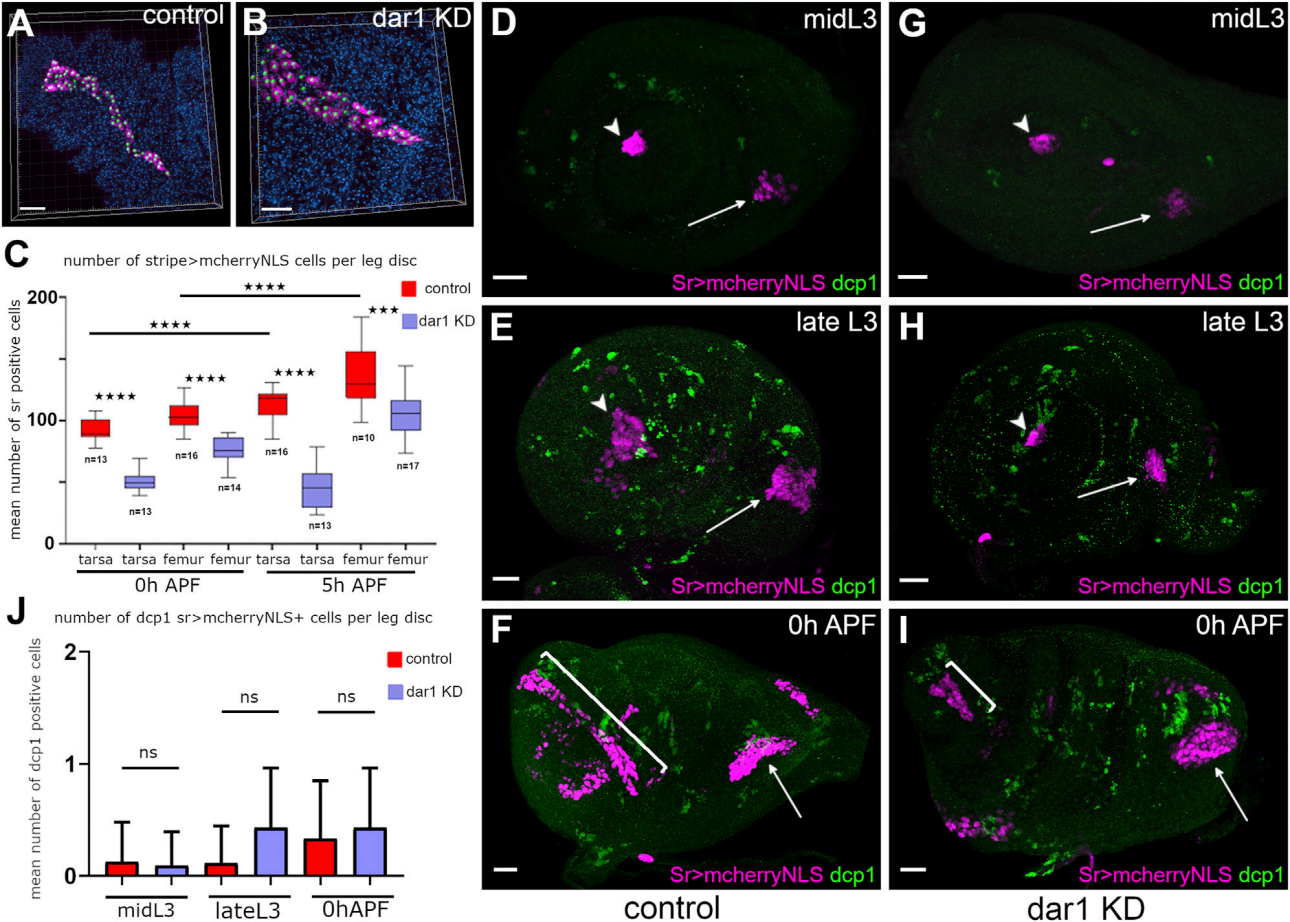
Reduction of tendon cell number in *dar1* KD leg discs is not due to ectopic apoptosis **(A,B)** examples of semi-automated counting tendon cells (magenta) from Z-stack projections of confocal images of lt from sr-gal4,dar1^3010/+^>UAS-mcherryNLS (A) and sr-gal4,dar1^3010/+^>UAS-mcherryNLS, UAS-dar1RNAi **(B)** 5 h APF leg discs, stained with DAPI (blue). Each green spot corresponds to a single cell **(C)** Box-plot diagram comparing number of mcherry-positive cells in lt and in the tilt, in control and in *dar1* KD leg discs at two different time points. Box boundaries and horizontal bar indicate 25/75 percentile and mean value, respectively. ***p < 0.001 and ****p < 0.0001 (Bonferroni or Welch test) **(D–I)** Confocal images of sr-gal4,dar1^3010/+^>UAS-mcherryNLS (magenta) **(D–F)** and sr-gal4,dar1^3010/+^>UAS-mcherryNLS,UAS-dar1RNAi **(G–I)** leg discs immunostained with anti-dcp1 (green) at different times of development. Arrowheads and arrows point to tarsal lt and tilt in femur, respectively. In both control **(D)** and *dar1* KD **(G)** L3 leg discs, cell death cannot be detected in lt or tilt. At late L3, several apoptotic cells are observed throughout the leg discs **(E,H)** but none of them are tendon cells. As stated previously, at this time there are already fewer tendon cells in the *dar1* KD leg disc **(H)** than in the control **(E)**. At early metamorphosis, lt (bracket) appears much shorter in the *dar1* KD leg disc **(I)** than in the control **(F)**, but no specific cell death is observed in this tendon **(J)** Graphs showing the mean number of sr > mcherryNLS cells that are dcp1positive per disc at different stages of development in control and *dar1* KD leg discs (total number of analysed discs, control *n* = 23, *dar1* KD *n* = 25). Scale bar 20 μm.

Although it has been shown that the number of cells that make up a tube is not closely correlated to tube length (Beitel and Krasnow, 2000), the question arose of whether the total number of sr-gal4 positive cell was affected after *dar1* KD. To find out, we counted the number of cells constituting both tarsal and dorsal femur tendons in sr-gal4,dar^3010/+^>UASmcherryNLS control pupae, and in sr-gal4,dar^3010/+^>UASmcherryNLS, UASdar1RNAi pupae at early stages of metamorphosis (Figures 6A–C). First, in control leg disc we saw a significant increasing number of sr-gal4>mcherryNLS cells from 0 to 5 h APF for both tendons, indicating that during the elongation process new Sr-positive cells were recruited. Next, we found that the number of sr-gal4>mcherryNLS cells was significantly reduced in *dar1* KD leg discs at the two different time points compared to the control leg disc. (Figures 6A–C). Lastly, in *dar1* depleted tarsal tendon, the number of sr-gal4>mcherryNLS cells was not statistically different between 0hAPF and 5hAPF, suggesting that no further tendon cells were recruited between these two time points, unlike in controls. Our results thus show that the failing of long tendon elongation due to *dar1* downregulation is associated to a defect of sr-gal4>mcherryNLS cell numbers. As a decrease in the number of cells may be due to a defect in cell proliferation and/or to an increase in cell death, we tested these two variables in sr-gal4,dar^3010/+^>UASmcherryNLS, UASdar1RNAi leg discs. Because Dar1 has been shown to restrict the proliferation of intestinal stem cells (Wu et al., 2018), we did not favor a defect of proliferation, still we immunostained *dar1* KD and control leg discs using phospho-histone three antibody. Although numerous mitotic cells can be observed in leg disc from mid-L3 stage (beginning of tendon cell induction) to early metamorphosis, only rare mitotic events could be observed among sr-gal4>mcherryNLS cells in this time window in both *dar1* KD and control conditions (Supplementary Figure S3).

To determine whether loss of sr-gal4>UASmcherryNLS cells could result from cell death increase, we performed immunostaining against caspase-activated dcp1 (Figures 6D–I). Between the time of induction of first Sr-positive cells and the early step of metamorphosis, we could barely detect sr-gal4>mcherryNLS apoptotic cells in *dar1* KD leg discs Figures 6M–O), while the defect in cell number was evident as early as the late L3 stage (compare Figures 6E,H). We confirmed this result by expressing GC3Ai fluorescent apoptotic sensor, which efficiently follows apoptotic cell dynamics tissue-specifically (Schott et al., 2017). Thus, when GC3Ai apoptotic sensor was specifically expressed in sr-gal4 cells, rare events of apoptosis were occasionally observed in both control and *dar1* KD leg discs (Supplementary Figure S4). We concluded that the up to 50% fewer tendon cells in *dar1* KD context could not be explained by any marked increase in cell death or a lower proliferation rate.

#### Epistatic Relationship Between dar1 and Stripe Expressions

**FIGURE 7:**
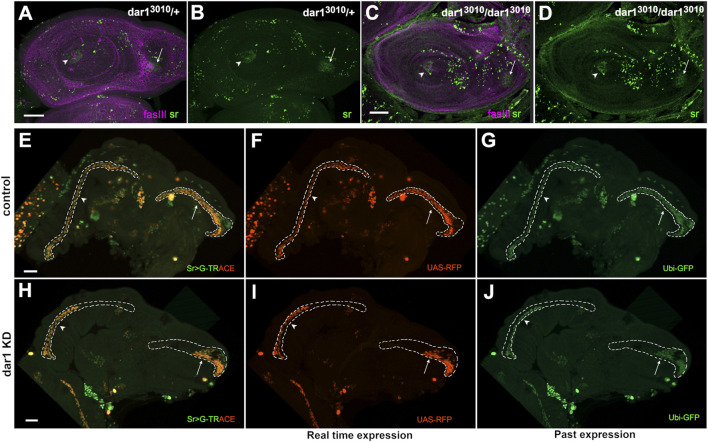
Dar1 is not required to initiate or maintain *stripe* expression **(A–D)** Confocal sections of L3 leg discs immunostained with anti-sr (green) and anti-fasIII (magenta) **(A–B)** In *dar1* ^*3010/+*^ heterozygous leg discs, *stripe* expression reveals tendon cell precursors in tarsal segments (arrowhead) and in dorsal femur (arrow) **(C–D)** In the leg disc of rare *dar1* ^*3010/3010*^ homozygous mutant escapers, *stripe* expression is still observed in tarsal segments (arrowhead) and dorsal femur (arrow) (total number of analysed discs, control *dar1* ^*3010/+*^ = 15, *dar1* ^*3010/3010*^ *n* = 12) **(E–J)** G-TRACE in sr-gal4,dar1^3010/+^ **(E–G)** and sr-gal4,dar1^3010/+^>UAS-dar1RNAi **(H–J)** leg discs at 5 h APF. RFP (red) staining shows current sr-gal4 expression (F, I) and GFP (green) reveals past expression **(G, J) (E,H)** merged G-TRACE expression. Dashed lines outline tarsal lt (arrowheads) and tilt in dorsal femur (arrows). At this stage, lt and tilt have deeply invaginated into the developing leg disc in the control **(E–G)**, with most of the tendon cells having maintained sr-gal4 expression (red and green staining). By contrast, in *dar1* KD leg discs **(H–I)**, the same tendons appear much shorter with both current sr-gal4 expression (red) and past sr-gal4 expression (green), indicating that shortening of those tendons is not due to premature loss of sr-gal4 expression in “missing” cells (extension of dashed lines in *dar1* KD prefigures the position where GFP positive cells should have been detected if the apparent tendon shortening was due to a premature loss of sr-gal4 expression). Scale bar 30 μm.

One obvious cause of missing tendon cells in the *dar1* KD leg disc would be a positive requirement of Dar1 to induce and/or maintain *stripe* expression (and so sr-gal4 expression). However, a direct requirement of Dar1 in inducing *stripe* expression is very unlikely because sr-gal4 driver comes from a Gal4 insertion in the *stripe* gene (sr-gal4^md710^) that is known to fairly recapitulate *stripe* expression (Soler et al., 2004; Usui et al., 2004), Indeed, all the phenotypes that we observed were obtained using sr-gal4 driver to induce UAS-dar1RNAi. So, if Dar1 was inducing *stripe* expression, we should expect a limited effect of dar1RNAi expression using this driver. Still, we decided to test this possibility by monitoring *stripe* expression in *dar1* ^*3010*^ homozygous null-mutant escapers at the time when initial pools of *stripe* expressing cells were set up in tarsi and dorsal femur (between early and mid-L3 larval stage). As expected, Stripe was still clearly detected at the L3 stage in both clusters *in dar1* ^*3010*^ heterozygous or homozygous mutants, indicating that *stripe* initial induction was very likely dar1-independent (Figures 7A–D). Conversely, expression of *dar1* was completely abolished when a dominant negative form of *stripe* was expressed in the leg disc epithelium (Supplementary Figure S5). These results strongly suggest that Stripe acts upstream to regulate *dar1* expression in the developing tendon, and that Dar1 is then needed to correctly pattern long tendons with the right number of Sr-positive cells. Finally, to determine whether the lack of Sr-positive cells in sr-gal4,dar^3010/+^>UASdar1RNAi context could be explained by a role for Dar1 in long-term maintenance of *stripe* expression, we performed a G-TRACE experimental analysis (Evans et al., 2009). This technique is used to determine the expression of a gal4 driver (here sr-gal4) at a current time point by inducing UAS-RFP expression (Figures 7F,I), and its historical expression by inducing UAS-Flippase expression. Flp enzyme recognizes FRT sites and removes the STOP cassette in the Act-FRT-STOP-FRT-GFP construct, allowing a permanent expression of GFP. Accordingly, if any cell has been committed as a sr-gal4 positive cell at any time during development and then loses this expression, it should still be detectable through sustainable GFP expression. In other words, RFP expression reveals real-time gal4 expression and GFP expression its past expression. In this way, we could see in both *dar1* KD and control 5 h APF leg disc, cells that currently expressed sr-gal4 (RFP+) are also GFP+ (Figures 7E,H). This result means that missing tendon cells in *dar1* KD disc are not cells that have lost their sr-gal4-positive fate. We therefore conclude that Dar1 is not required to maintain *stripe* expression in developing tendon cells.

Altogether, our results show that Dar1 is required for a proper morphogenesis of the long internal leg tendons. Thus, length shortening of the tendons in *dar1* KD context is associated to a dramatic decrease in Sr-positive cell numbers, which cannot be explained by cell death, proliferation defect or loss of *stripe* expression.

## Discussion and Perspectives

In *Drosophila* during metamorphosis, epithelium-derived progenitors of tendon legs undergo critical morphogenetic changes to adopt a unique tube-like structure for muscle attachment sites. Long tendon morphogenesis is characterized by several steps considered hallmarks of canonical tubulogenesis, such as cell apical constriction and tissue invagination, followed by collective cell migration (Maruyama and Andrew, 2012; Girdler and Röper, 2014; Hayashi and Kondo, 2018). Another mark of tubulogenesis is that developing long tendons form an apical lumen and display F-actin-rich protrusions at the basal membrane of migrating cells (Laddada et al., 2019). Our newly-generated transcriptomic data, specific to leg tendon precursors, are consistent with these observations. These data highlight the expression of genes related to cell-shape rearrangement and cytoskeleton regulation, echoing the deep cell rearrangement of these epithelium-derived cells. Corroborating the importance of the tubulogenic process in leg tendon morphogenesis, GO analysis highlighted GO terms such as tube size regulation and morphogenesis. We also validated the involvement of the tubulogenic process by recapitulating the phenotypes observed in other systems, such as the tube length defects observed when *serpentine* and *vermiform* expressions are knocked down in the tracheal system (Luschnig et al., 2006).

Tendons are crucial components of the musculoskeletal system but still our knowledge on the developmental program that controls tenogenesis remains poorly investigated, even though tendon injuries are very common. Identifying transcription factors and their roles in regulating this developmental program is crucial to develop new effective treatments for tendon repair. For instance, known transcription factors, such as Scleraxis, Mohwak and EGR1, associated with tendon development in vertebrates are also activated upon tendon injury (reviewed in Nourissat et al., 2015). So, to pinpoint critical transcriptional regulators of leg tendon development, we performed a short lethality and climbing RNAi screen. Our gene preselection, based on our transcriptomic data, was especially helpful: nearly 65% of these candidates induced locomotion defect and/or lethality when downregulated in tendon cells, even though RNAi knockdown efficiency is often limited by residual gene expression (Perkins et al., 2015). In comparison, a previous unbiased RNAi screen targeting 1,384 genes to uncover genes involved in flight muscle attachment sites produced only about 1.5% positive candidates (Tiwari et al., 2015). Thus, the efficiency of our RNAi screen also emphasizes our RNA-seq data reliability.

Among the newly-identified candidates, we found that tendon expression of *dar1* was restricted to the appendicular long tendon and was not found in other tendon precursors that do not form long internal structures (tendons of flight muscles or larval muscle tendon), making this gene an excellent candidate for the specific development of long tendons. Correlating climbing defects, the LOF of this gene impaired the formation of the leg long tendon. *dar1* KD led to a clear-cut tendon phenotype by the end of larval stages and early metamorphosis when long tendons are specified and when they elongate. Our further analysis of *dar1* function shows that Dar1 participates in the cellular events required for actin cytoskeleton organization and specifically filopodia formation. It also highlights a critical role for this Krüppel-like factor in the elongation of the long tendons. Although there is no clear evidence for a direct role of filopodia in the tubulogenesis process, migrating cells form two types of actin-based protrusions, the lamellipodia, involved in cell motility, and the filopodia, which may sense and interact with the surrounding environment (Svitkina et al., 2003; Girdler and Röper, 2014; Okenve-Ramos and Llimargas, 2014). Thus, although our work does not show that cell tendon shortening is a direct consequence of the decrease in filopodia numbers, it makes of Dar1 an interesting potential link between actin-rich protrusion formation and tube elongation. Interestingly, *dar1* has been shown to regulate the dendritic microtubule cytoskeleton by suppressing the expression of the microtubule-severing protein spastin (Ye et al., 2011), which we found depleted in our RNAseq data. So, we have tried to phenocopy *dar1* KD by overexpressing *spastin* in tendon cells, but it systematically and rapidly induced death of the larvae, which unabled us to clearly elucidate whether manipulating spastin activity could indirectly affect filopodia formation in developing tendons. Nevertheless, reciprocal interactions between MT and actin networks is a well-established paradigm (Rodriguez et al., 2003; Mohan and John, 2015). For instance, the growth cone in neuronal cells depends on coordinated interactions between MTs and actin filaments (Schaefer et al., 2002; Geraldo and Gordon-Weeks, 2009; Bearce et al., 2015). We therefore cannot exclude the possibility that defects in actin organization observed upon *dar1* downregulation are due to alterations of MT assembly and/or MT-actin network coordination. Supporting this last hypothesis, comparison of gene expression profiles between midgut cells expressing *dar1* RNAi and overexpressing *dar1* showed a deregulation of *DAAM* expression (Wu et al., 2018). *DAAM* encodes a formin known to link and coordinate actin and microtubule network dynamics (Szikora et al., 2017). It would thus be of interest to unravel a potential regulation of Dar1 on DAAM, or more broadly, to perform a Dar1 genetic screen interaction with actin and microtubule modulators.

Concomitantly to the defect in long tendon growth and actin cytoskeleton organization, we demonstrated Dar1 involvement in recruitment of new Sr-positive cells. First, we found that additional tendon progenitors were recruited, after initial expression of *stripe* in L3, contributing to the development of long tendons during their elongation in a normal context. Secondly, our finding showed that *dar1* KD led to a reduction of the number of Sr-positive tendon progenitors without apparent effect on apoptosis and the number of mitotic cells suggesting that *dar1* might control *stripe* expression (by inducing or maintaining its expression). However, our lineage and epistasis experiments indicate instead that Dar1 acts downstream of Stripe and that Stripe is necessary for inducing *dar1* expression. We can thus infer that Stripe regulates tendon morphogenesis through *dar1* activation, which is in turn responsible for tendon elongation but also necessary to recruit new Sr-positive cells in an undetermined mechanism. Organ morphogenesis often correlates with cell-fate induction, and numerous studies in the last decade have shown how multicellular morphogenetic events can feed back into gene regulatory pathways to specify cell fate (Chan et al., 2017). Thus, during the 3D changes of a pseudo-stratified epithelium, the polarity and cytoskeleton rearrangements of the engaged cells are sensed by the neighboring cells through mechano-transduction. For instance, tissue deformations caused by germ-band extension upregulate the expression of the transcription factor twist via nuclear translocation of *β*-catenin (Desprat et al., 2008). We had previously shown that the Notch pathway is required for the initial expression of *stripe* in L3 stages (Laddada et al., 2019). However, conditional depletion of Notch activity at a later time point (early metamorphosis) did not appear to alter dorsal femur long tendon formation, whereas we show here that recruitment of additional Sr-positive tendon precursors is still ongoing at this time. It is tempting to speculate that morphological changes, partly driven by *dar1*, could be mechano-transduced to the following cells and activate *stripe* expression in an alternative Notch-independent way. Alternatively, local reorganization of the leg disc epithelium by the primary invagination of first specified Sr-positive cells could also play a role by exposing the surrounding cells to local signals. Kesavan et al. have demonstrated the role of CDC42-mediated tubulogenesis as a driver of non-cell-autonomous cell fate specification in pancreatic tubulogenesis (Kesavan et al., 2009). They showed how this small RhoGTPase, through its role in tube formation as an actin nucleator, provided the correct microenvironment for proper cell specification. Our own results indicate *dar1* KD impacts both tendon elongation and number of tendon cells. Further work is required to determine whether cellular rearrangement during tube formation can have an impact on the recruitment of new tendon progenitors. For instance, it could help determining whether Dar1 transcriptional activity regulates major actors of the cytoskeleton organization in order to trigger a spatial rearrangement of the epithelial cells. Such reorganization could provide a permissive microenvironment for a specifying signal that remains to be identified.

Although animal appendages have long been seen as non-homologous structures, there is growing molecular evidence that limbs of vertebrates and insects could arise from an ancestral appendage developmental program (Pueyo and Couso, 2005). Recently, Tarazona et al. reported that genes and signaling pathways that guide the development of both vertebrates and arthropods also control the development of cephalopod mollusk arms and tentacles (Tarazona et al., 2019). Their results thus strongly suggest that bilaterian appendages evolved by parallel activation of a genetic program that was present in a common ancestor. Whether the development of limb internal structures follows a similar conserved path is less well-documented. We have previously shown that the key regulator of vertebrate appendicular myogenesis, the *lb/Lbx1* gene, is also required for proper leg muscle identity in *Drosophila* (Maqbool et al., 2006). Strikingly, Huang et al. have shown that long tendons in the mouse form by a rapid elongation of the tendon in parallel with skeletal growth, and that this elongation is fueled by the recruitment of new mesenchymal progenitors (Huang et al., 2019). This recruitment is dependent on the transcription factor Scleraxis (Scx), which appears to be unnecessary for first tendon anchoring but is subsequently needed for the recruitment of new progenitors during tendon elongation. Although the *Scx* ortholog has not yet been identified in *Drosophila,* our study identified transcription factor Dar1 as a specific factor of elongating tendons in *Drosophila*. Interestingly, its mammalian counterparts KLF5 and KLF4 have also been identified in two independent transcriptomic analysis of mouse limb tendon cells (Havis et al., 2014; Liu et al., 2015) and expression of KLFs have been reported in connective tissue surrounding tendon cells in the chicken (Orgeur et al., 2018). More strikingly, a recent study (Kult et al., 2021) showed that tendon-to-bone attachment cells have a bi-fated origin, with the ability to activate a combination of chondrogenic and tenogenic transcriptomes, and those authors identified KLF2/4 as central regulators of these unique bi-fated cells in vertebrates. This work and our previous results (Laddada et al., 2019) indicate that Dar1/KLF-positive cells, which shape the leg long tendons, originate from tendon precursors (Sr-positive cells) that are selected among the cells of leg segmental joints expressing odd-skipped. Thus, our most recent findings and those of others suggest an evolutionary conserved function of KLFs in the complex integration of the musculoskeletal system of the limb.

## Materials and Methods

The following *Drosophila* stocks were used: R10H12-gal4 (Pfeiffer et al., 2008; BDSC 48278), UAS-mCherryCAAX (BDSC 59021), UAS-mCherryNLS (BDSC 38425), enhancer trap lines sr-gal4^md710^ (Usui et al., 2004, BDSC 2663), UAS-Lifeact.GFP (BDSC 35544), UAS-dar1RNAi (BDCS 31987), *dar1* ^*3010*^ (BDSC 65269), UAS-GC3Ai (Schott et al., 2017, BDSC 84346), UAS-serpRNAi (BDSC 63556), UAS-vermRNAi (BDSC 57188), UAS-lolalRNAi (BDSC 35722), UAS-Dicer2 (BDSC 24650), UAS-SrDN (gift from Vorbrüggen and Jäckle, 1997) and G-TRACE line (Evans et al., 2009, BDSC 28280).

### Cell Sorting and RNA Extraction

The fluorescence-activated cell sorting (FACS) protocol was adapted from (Harzer et al., 2013). Briefly, approximately 50 UAS-Lifeactin.GFP; sr-gal4/TM6b, tb white pupae (0 h APF) were dissected on ice to collect 250–300 leg imaginal discs in M3 (S3652 Sigma Aldrich) complemented medium. Leg disc cells were dissociated in collagenase (P4762-Sigma Aldrich) and papain (C267-Sigma Aldrich) solution for 1 h at 30°C, 300 rpm (Thermomixer Eppendorf), with additional mechanical stirring. After filtering, we used a FACSAriaTM device (4 °C, 20 psi, nozzle ∅ 100 μm) to first sort a batch of cells (roughly 50,000 cells per sample) independently of fluorescence, which we used as input (IP) and we then sorted the tendon cell (GFP+) population based on GFP fluorescence. Samples were directly collected in Trizol reagent (Invitrogen, 15596026). RNA extraction was performed with Zymo Quick-RNA microprep kit (R1051), sample RNA quality and quantity were estimated with QuBit (ThermoFisher, Qubit RNA HS Assay Kit, Q32852) and BioAnalyzer (Agilent, Agilent RNA 6000 Nano Kit 5067-1511), and sample specificity was analyzed by qPCR. Samples were stored at−80°C. For the detailed protocol, see Supplementary Material S1.

### RNAseq and Transcriptomic Data Analysis

Extracted total RNA from GFP+ and IP cells from three different replicates were sent to Heidelberg genomic platform EMBL for high-throughput mRNA sequencing (NextSeq 500/Illumina). They generated mRNA libraries with NEB RNA Ultra kit (New England Biolabs E7770 L) and dToligos probe were used to target mRNA for cDNA synthesis. Single-end multiplex was performed. Bioinformatic analysis was performed by Dr Yoan Renaud using FastQC and Bowtie2 software (reference genome Dm6). FPKM and differential expression between IP and GFP + samples were determined using R script DEseq2 software. Sample correlations are given in Supplementary Material. Differential expression was performed using R-package DEseq2. Fold change (FC) between GFP+ and IP samples was computed using their normalized raw counts. GEO accession number: GSE169313.

### *In Vivo* RNAi Screen

53 UAS-RNAi lines (see Supplementary Table S2) from two separate stock centres, VDRC and BDSC (Dietzl et al., 2007; Ni et al., 2009) were crossed with UAS-dicer2, UAS-CAAXmcherry; sr-gal4/TM6b,tb, hu line and screened for lethality and/or climbing defect. 5–10 males carrying UAS-RNAi were mated with 15–20 females. Crosses and egg laying were performed at 25°C; 48 h after egg laying, larvae were transferred at 28°C.

Embryonic or early larval stage lethality rate was measured by counting the number of non-tubby over tubby third instar larvae. Percentage of lethality was calculated by applying formula (1 − (number of non-tubby larvae)/(number of tubby larvae)) × 100. Crosses were considered as positive for embryonic (early larval stage) lethality when lethality was higher than the 20% arbitrarily chosen threshold. To assess the percentage of lethality during metamorphosis, we applied formula (1 − (the number of non-tubby, non-humeral emerging adult flies)/(total number of initial non tubby pupae)) × 100. Crosses with a higher score than 20% were considered positive candidates for metamorphosis lethality.

When sr-gal4>UAS-RNAi adult flies survived, we performed a climbing test adapted from the RING (Rapid Iterative Negative Geotaxis) assay published by Gargano et al. (Gargano et al., 2005). Up to ten young non-balanced flies (48 h old) were transferred to an empty vial and allowed to recover at room temperature for at least 2 h. The vial was then rapped sharply on a table three times in rapid succession to initiate negative geotaxis responses. The flies’ positions in the vial were digitally captured after 5 s and we calculated the percentage of flies remaining in the bottom third of the vial. For each cross, two replicates (two vials of ten flies) were measured three times. We calculated the mean of these six trials. When this mean was equal to or greater than 30%, the corresponding cross was scored as “climbing defect”.

RNAis directed against *stripe* (100% embryonic lethality) and mcherry (0% embryonic lethality, 5% metamorphosis lethality, 15% climbing defect) were considered as positive and negative controls, respectively.

### Immuno-Histochemistry, Cryosections and Confocal Microscopy

The following primary antibodies were used: guinea pig anti-Dar1 (1:250, courtesy of B. Ye), guinea pig anti-Stripe (1:500, from T. Volk), mouse anti-Fas III (1:500, Developmental Studies Hybridoma Bank [DSHB]), rabbit anti-Twist (1:500, our lab), mouse anti-Dlg (1:500, DSHB), chicken anti-GFP (1:500, Abcam), rat anti-DE-cadherin (1:500, DHSB), rabbit anti-Dcp1 (1:100, Cell Signaling), rabbit anti-pH3 (1:1000, Invitrogen), and anti-mcherry (rabbit, Abcam). Muscle fibers were visualized using cy3 or cy5-conjugated phalloidin (1:1000, Invitrogen) and nuclei using DAPI (ThermoFisher D1306). Secondary antibodies (Jackson) anti-rabbit, anti-guinea pig, anti-chicken and anti-mouse conjugated to Alexa488, cy3 or cy5 fluorochromes were used (1:500). Immunohistochemistry experiments were performed on samples fixed in 4% paraformaldehyde (PFA) for 20 min, rinsed in 0.1% PBS-Triton and blocked in 10% horse serum before immunostaining with primary antibodies (at 4°C, overnight). The samples were then rinsed and incubated with appropriate secondary antibody (at room temperature, 1 h). For cryosections, we dissected adult leg and fixed them from 40 min to 1 h in 4% PFA. Fixed samples were then incubated in sucrose solution (30%) at 4 °C overnight. Adult legs were then laid on the bottom of a plastic well and carefully covered with Neg-50TM gel (Richard-Allan Scientific). The preparation was frozen at −80°C to set the gel. Sections were cut with a cryostat at 4°C (thickness 18–20 um). The sliced samples were stained with phalloidin before imaging. Immunostaining was visualized on an inverted SP8 Leica confocal microscope, and images were analyzed with Imaris 7.6.5 software.

### Tendon Length Measurement

Tendon size was determined by normalizing the length of the tendon over the whole size of the imaginal disc. For this purpose, we used the bounding-box function of the Imaris software to semi-automatically generate a 3D mesh of the imaginal disc (using FasIII staining). The disc was viewed as an ellipse with major axis (length) *X* and minor axis (width) *Y*. Discs were considered as flat objects, since at the developmental stages of analysis the thickness (*Z*) dimension has no significant impact on the measurement. From the ellipse-disc we computed the radius *R* of each corresponding disc (*R* = √*X* × *Y*⁄2). The lengths of tendons were measured using the function *MeasurementPoint* by manually following their pathways. Finally, we normalized the tendon length by dividing the length by the disc radius *R*. Statistical analyses were carried out using Prism software with an analysis of variance (ANOVA) between the different conditions and the different stages. Statistical significance between control and knockdown conditions was estimated using the Bonferroni test.

### Counting Cell Number in Developing Tendon

0 and 5 h APF leg discs from sr-gal4,dar1^3010/+^>UAS-mcherryNLS and sr-gal4,dar1^3010/+^>UAS-mcherryNLS,UAS-dar1RNAi pupae were dissected and stained with DAPI for 1 h. 0.5 μm confocal sections of long tendon of tarsi and dorsal tendon in femur (tilt) were performed. Images were processed with Imaris software and numbers of mcherryNLS and DAPI-positive cells per tendon were determined automatically using the function *Spots*. The automatic cell counting was subject to manual adjustement.

Statistical significance was estimated using the Bonferroni or Welch test.

## Data Availability

The datasets presented in this study can be found in online repositories. The names of the repository/repositories and accession number(s) can be found below: https://www.ncbi.nlm.nih.gov/geo/, GSE169313.
