# Supplementary material for: Transcriptomic and Genetic Analyses Identify the Krüppel-Like Factor Dar1 as a New Regulator of Tube-Shaped Long Tendon Development
Source: Front Cell Dev Biol. 2021 Dec 16;9:747563. doi: 10.3389/fcell.2021.747563 (PMC8716952; doi:10.3389/fcell.2021.747563)
Supplement: Supplementary file 1 [file Table1.pdf]

Flybase ID	Gene names	Observed phenotype
FBgn0001316	klar	no observed phenotype
FBgn0085044	mew/PS1*	occasional tendon shortening/locomotion defect
FBgn0000308	chic	lethality at early pupal stage
FBgn0010333	Rac1*	strong larval lethality (UAS-rac1 DN), clusters of tendon cells disorganized
FBgn0014010	Rab5*	strong larval lethality (UAS-rab5 DN), clusters of tendon cells disorganized
FBgn0022238	lolal	locomotion defect, excessive tendon elongation
FBgn0267348	LanB2	no observed phenotype
FBgn0261341	Verm	pupal lethality, excessive tendon elongation
FBgn0260653	Serp	locomotion defect, excessive tendon elongation
FBgn0085148	If (PS2)*	pupal lethality, occasional tendon shortening
FBgn0004657	Mys (betaPS)*	most flies die in pupae, escapers show strong locomotion defect with most leg tendon discrupted

Supplemental Table S1. List of tested genes involved in morphogenetic processes.
UAS-RNAi lines for selected candidates or alternatively UAS-Dominant Negative line were crossed with sr-gal4>UAS-Lifeact.GFP and, when required, combined with gal80ts allele to by-pass embryonic lethality (genes with asterisk).
