# Supplementary material for: Transcriptomic and Genetic Analyses Identify the Krüppel-Like Factor Dar1 as a New Regulator of Tube-Shaped Long Tendon Development
Source: Front Cell Dev Biol. 2021 Dec 16;9:747563. doi: 10.3389/fcell.2021.747563 (PMC8716952; doi:10.3389/fcell.2021.747563)
Supplement: Supplementary file 2 [file Table2.pdf]

1.Genes	2.Flybase IDs	3.library	4. ID stock	5. embryonic/early larval lethality (%)	6. pupal lethality (%)	7. % of flies showing climbing defect	8. FPKM	9. Fold Changed (FC)
CG15435	FBgn0031608	VDRC<br>TRiP	100074<br>65064	0<br>41	10<br>51	23,33<br>53,33	13,4316	1,52
CG3163*	FBgn0034961	VDRC	45767	18	0	<10%	29,1401	1,57
CG5641	FBgn0038046	VDRC<br>TRiP	48200<br>56895	0<br>3	5<br>6	<10%<br>11,11	126,366	1,65
CG6276	FBgn0038316	VDRC<br>TRiP	30141<br>27064	9<br>0	27<br>36	<10%<br>50,00	29,6684	1,56
CG9650	FBgn0029939	TRiP<br>VDRC	26713<br>104402	0<br>0	22<br>0	60<br>90,4	18,2011	1,6
CG9948*	FBgn0035721	VDRC	29429	10	4	40,00	16,4959	1,53
Dichaete	FBgn0000411	VDRC<br>TRiP	107194<br>26217	52<br>10	9,52<br>19	33,33<br>23	17,5199	1,56
e(y)2	FBgn0000618	VDRC<br>TRiP	16751<br>42524	0<br>26	3<br>40	20<br>20,00	40,8249	1,68
HP1c	FBgn0039019	VDRC<br>TRiP	104893<br>31339	4<br>0	11<br>7	<10%<br>20,00	118,756	1,55
ladybird early (lbe) / lbl	FBgn0011278	VDRC<br>TRiP	12662<br>60001	0<br>7	13<br>1	<10%<br><10%	74,5073	1,51
Nf-YB	FBgn0032816	VDRC<br>TRiP	103655<br>57254	0<br>0	6<br>10	30<br>46,67	32,1098	1,61
REPTOR-BP	FBgn0032202	VDRC<br>TRiP	19110<br>29542	3<br>0	8<br>32	16,67<br>36,67	36,6661	1,65
sox15	FBgn0005613	VDRC<br>TRiP	45482<br>57264	2<br>18	15<br>7	30,00<br>50,00	20,3571	1,65
CG17803	FBgn0038547	VDRC<br>TRiP	38869<br>60022	0<br>0	0<br>24,55	<10%<br>3,33	23,1472	3,71
drm	FBgn0024244	VDRC<br>TRiP	1020405<br>42548	0<br>0	45<br>1	100,00<br><10%	405,8470	4,1
E(spl)mgamma-HLH	FBgn0002735	VDRC<br>TRiP	10950<br>25978	0<br>9	0<br>26,81	<10%<br><10%	64,4192	2,11
dar1	FBgn0263239	VDRC<br>TRiP	26980<br>31987	0<br>0	0<br>62	73,30<br>90,90	33,5114	4,7
Dr*	FBgn0000492	TRiP	42891	0	0	<10%	31,8571	
odd	FBgn0002985	VDRC<br>TRiP	102405<br>28295	0<br>0	45<br>0	100,00<br><10%	28,3636	2,42
Sox14*	FBgn0005612	TRiP	34794	0	0	<10%	27,9784	1,72
CG7785	FBgn0038564	VDRC<br>TRiP	109307<br>57715	0<br>0	27,64<br>31	20<br>46,70	16,6135	1,8
CG11317*	FBgn0039816	TRiP	28065	100	nd	nd	16,2672	5,5
Dlip1	FBgn0040467	VDRC<br>TRIP	11245<br>57717	0<br>48	0<br>1,67	<10%<br>3,33	15,5048	1,91
ato	FBgn0010433	VDRC<br>TRIP	2924<br>34929	6<br>0	0<br>8,27	<10%<br>42,42	15,4854	5
E(spl)m5-HLH	FBgn0002631	VDRC<br>TRiP	47124<br>26201	18<br>0	14<br>0	<10%<br><10%	14,1928	1,8
toe*	FBgn0036285	TRiP	50660	0	0	<10%	13,7040	2,28
CG44247*	FBgn0265182	VDRC	7413	0	0	<10%	13,5124	5,12
Sidpn*	FBgn0032741	VDRC	3065	0	0	<10%	12,6620	4,95
esg*	FBgn0001981	TRiP	28514	18	28	100,00	101,44600	1,8
l(2)k10201	FBgn0016970	VDRC<br>TRiP	108881<br>57548	17<br>39	54,69<br>12	<10%<br>13,33	10,262	2,1
Stripe	FBgn0003499	VDRC<br>TRiP	105282<br>27701	100<br>100	nd<br>nd	nd<br>nd	54,4971	7,89
mCherry*		TRIP	35785	0	5	15,00	nd	nd

* no alternative (VDRC or TRIP) availbale stock

Supplemental Table S2. List of the 31 analyzed transcription factors and RNAi screen data.

Columns 1 and 2 show gene names and Flybase IDs, respectively. Columns 3 and 4 show stock origin of tested UAS-RNAi lines and ID stock number, respectively. Column 5 shows the percentage of lethality in embryo or early larval stages induced by UAS-RNAi line expression using sr-gal4 driver. Crosses showing a lethality higher than the set threshold of 20% were considered positive. Column 6 shows the percentage of lethality during metamorphosis. Crosses showing metamorphosis lethality higher than 20% were considered as positive. Column 7 indicates the percentage of adult flies showing climbing defect. We set the threshold of 30% above which RNAi lines were considered positive. For a detailed description of lethality and climbing screen, see Materials and Methods. Columns 8 and 9 indicate transcript FPKM (Fragments Per Kilobase Million) and Fold Change expression in GFP+ cells compare to whole leg disc cells (p-value<0,05), respectively.
