# Supplementary material for: Transcriptomic and Genetic Analyses Identify the Krüppel-Like Factor Dar1 as a New Regulator of Tube-Shaped Long Tendon Development
Source: Front Cell Dev Biol. 2021 Dec 16;9:747563. doi: 10.3389/fcell.2021.747563 (PMC8716952; doi:10.3389/fcell.2021.747563)
Supplement: Supplementary file 3 [file DataSheet1.DOCX]

**Supplementary Material and Data**

**FACS** **detailed protocol**

1. **Dissection**

- Prepare sterile filter solution of 50 ml M3 medium (S3652 Sigma Aldrich) complemented with 50 μl l-glutathion (G6013 Sigma Aldrich, 0.04 mg/ml final), 1.5 ml l-glutamine (G7513 Sigma Aldrich 100 ml), 250 μl 2% penicillin/streptomycin solution (P4458 Sigma Aldrich 50X). The solution can be stored for 1 week at 4 °C.
- Prepare between 50 to 60 0h pupae with genetic background of interest. Wash three times in PBS/EtOH (30/70) and once in PBS (1X) to remove food and impurities.
- Dissect on ice, for 1 h maximum in M3 complemented medium to collect around 250–300 leg imaginal discs.

1. **Dissociation and cell sorting**

- Prepare dissociation mix: 450 ul M3 complemented medium plus collagenase 20 mg/ml (P4762-Sigma Aldrich) 25 μl, papain 20mg/ml (C267-Sigma Aldrich) 25 μl.
- Transfer leg imaginal disc to 1.5 ml tube. This step is critical and must be done slowly to avoid tissue wasting, we recommend using a PBS-coated Pasteur pipette.
- Add the dissociation mix and perform mechanical dissociation on thermomixer (30 °C, 1 h, 300 rpm) and gentle mechanical stirring every 15 min using a pipette.
- Carefully filter the cell suspension using FlowmiTM Cell strainers (40 μm) in hemolysis tubes. Rinse the filter with 500 ul of M3 complemented medium. Keep on ice before cell sorting.
- Collect cells (4°C, 20 psi, nozzle ∅ 100 μm) directly in Trizol. Samples can be stored at −80 °C before RNA extraction.

1. **RNA extraction**

- Add 100 μl of chloroform, agitate vigorously and let mixture stand for 3 min at room temperature.
- Centrifuge at 12,000 rpm for 15 min at 4 °C.
- Carefully collect the supernatant.
- Precipitate the RNA in 0.5 × volume of 100% cold isopropanol at RT.
- Transfer the solution on a Zymo Quick-RNA microprep column and perform RNA extraction following the manufacturer’s instructions
- Elute with 12 ul RNAse-free water pre-warmed to 42 °C.
- Perform RNA quantitative analysis using Qubit (ThermoFisher, Qubit RNA HS Assay Kit, Q32852) and quality assay using Bioanalyzer (Agilent, Agilent RNA 6000 Nano Kit 5067-1511). Store samples at −80 °C.

**qPCR analysis**

qPCR analysis has been performed to control sample specificity using the following primers:

Rp49

Fw GCTTCAAGGGACAGTATCTG

Rv AAACGCGGTTCTGCATGAG

SrB1 3-4

Fw CGACCTCAACACACCGGT

Rv GGGGTTCCAAAGACAGATCC

SrB2 3-4

Fw CAGCTGATCGAGGCGCTG

Rv TCCAAAGACAGATCCTCGGA

eGFP

Fw GGAGCGCACCATCTTCTTCA

Rv AGGGTGTCGCCCTCGAA

gal4DBD

Fw GCATGCGATATTTGCCGACTTAAAA

Rv TCCCAGTTGTTCTTCAGACACTTG

twist

Fw AAGTCCCTGCAGCAGATCAT

Rv CGGCACAGGAAGTCAATGTA

Dll 2-3

Fw GAACTCCTACTCCGGCTACC

Rv GAGTCCTCGCACTTATCGGA

Prospero

Fw ACGGCATGGCTCCTACTTCT

Rv AGAACATCAGCTTGGCCTTG


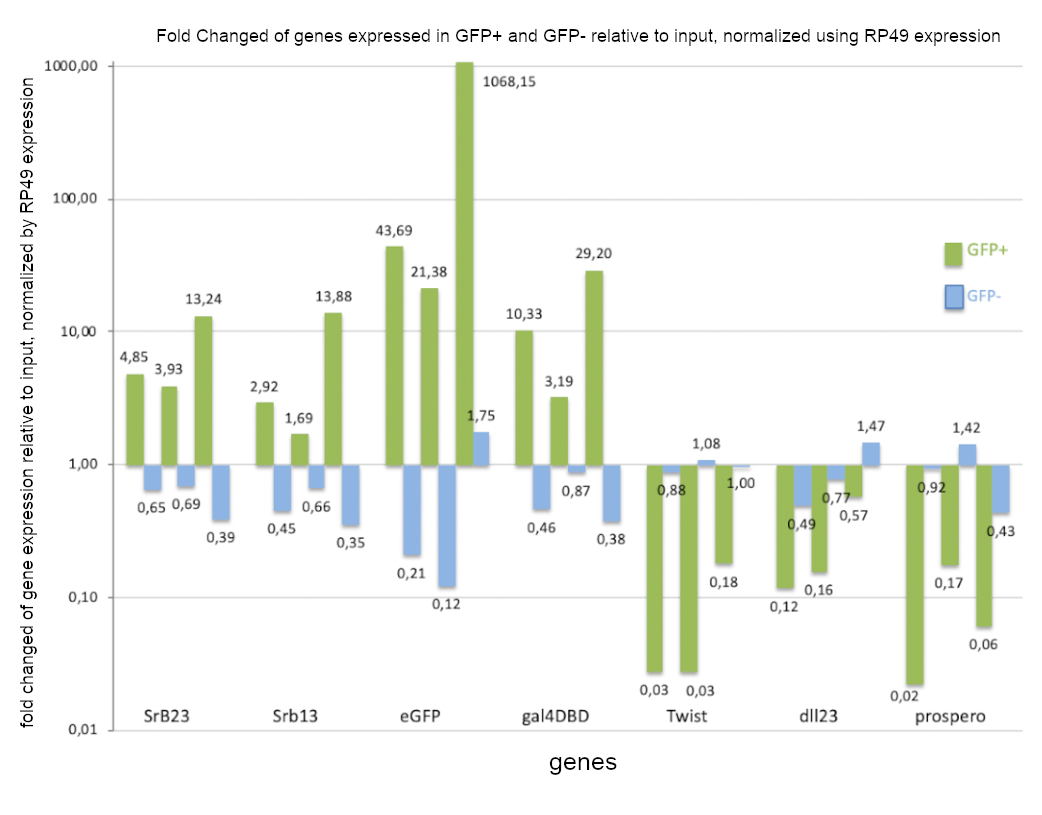


**Pearson correlation between three independant RNAseq replicates:**

(IP= input)

**
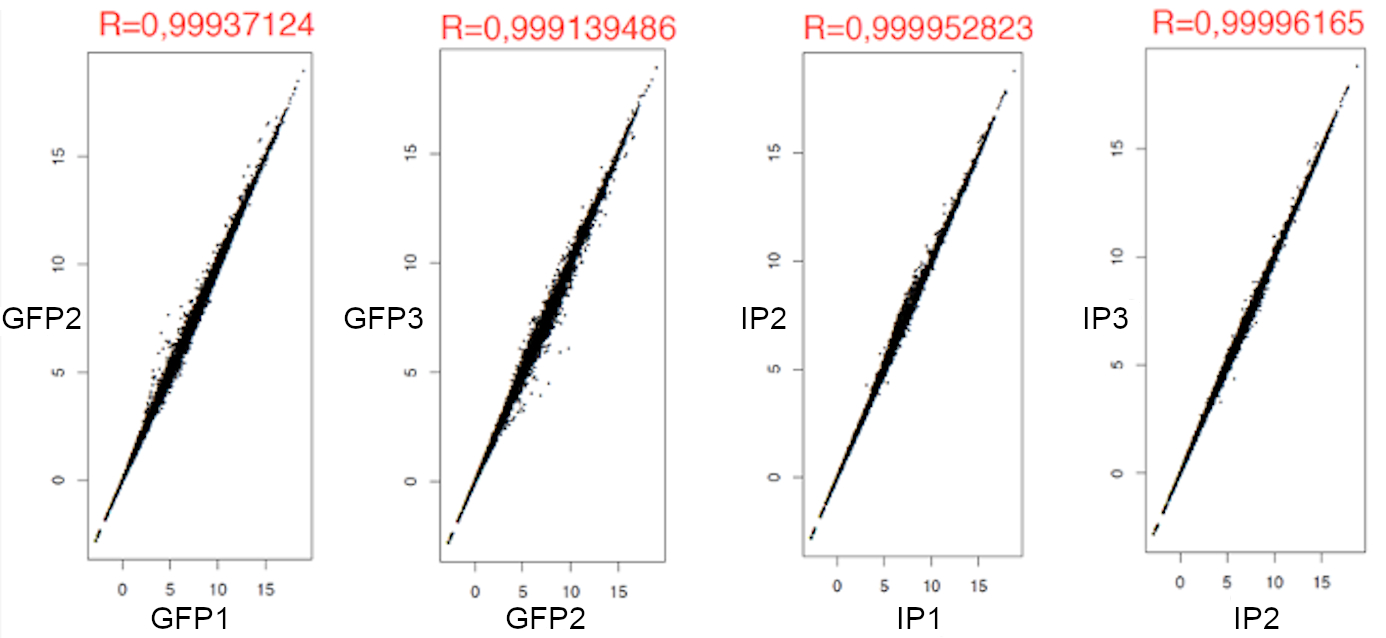
**
