# Supplementary material for: Transcriptomic and Genetic Analyses Identify the Krüppel-Like Factor Dar1 as a New Regulator of Tube-Shaped Long Tendon Development
Source: Front Cell Dev Biol. 2021 Dec 16;9:747563. doi: 10.3389/fcell.2021.747563 (PMC8716952; doi:10.3389/fcell.2021.747563)
Supplement: Supplementary file 4 [file Image1.pdf]

Supplemental Figures

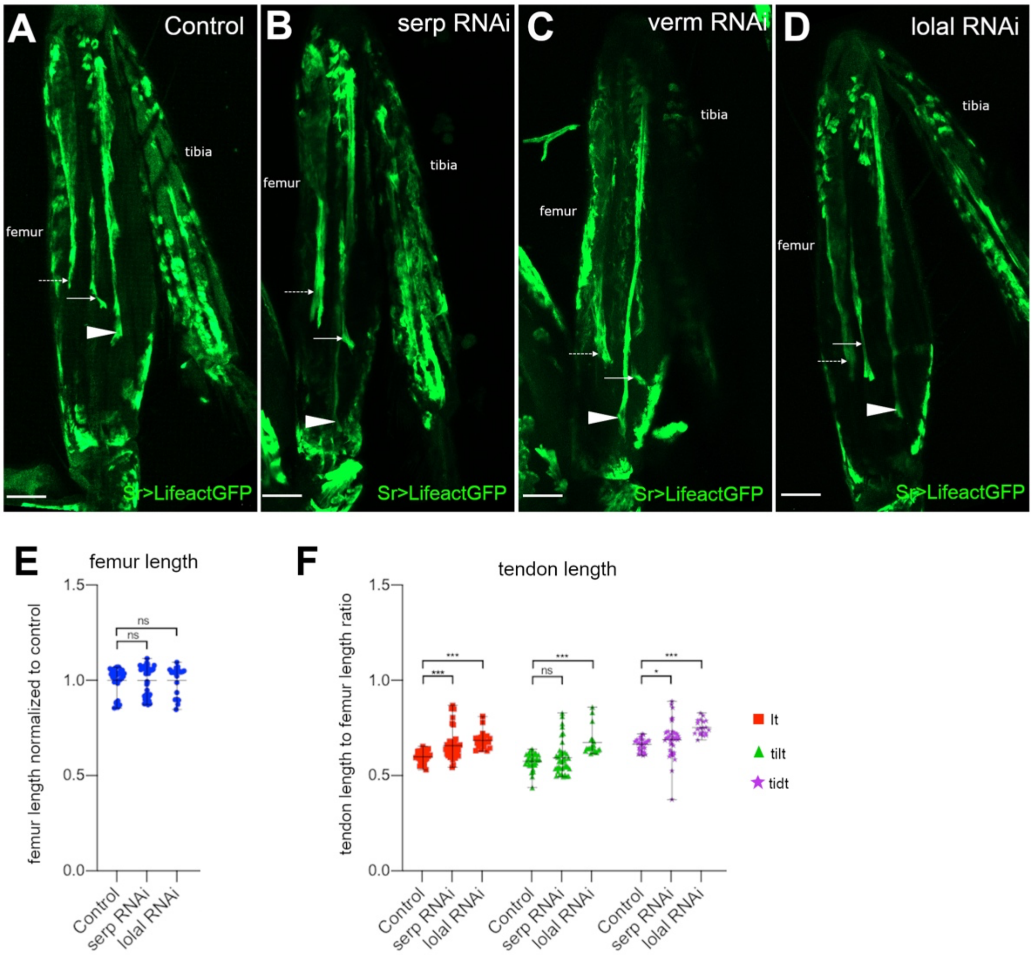

Supplemental Fig. 1. Downregulations of tube-related genes, serpentine, vermiform and lolal affect tendon elongation.

(A-D) Confocal sections of sr-gal4>UAS-Lifeact.GFP femur adult legs. To facilitate our investigation, we focused on the femur segment where three representative long tendons are easy to distinguish: the tibia depressor tendon (tidt) in ventral position (arrowhead), the tibia levator tendon (tilt) in dorsal position (dotted arrow) and the long tendon (lt) of tarsi (arrow). This last runs from the distal tip of the leg and terminates in the mid-proximal femur (for a complete description and nomenclature, see Soler et al. 2004). Compared to the control (A), these three tendons appear visually more elongated when sr-gal4>UAS-Lifeact.GFP is crossed with UAS-RNAi lines against serp (B), verm (C) and lolal (D). (E) Dot-plot graph showing mean of femur length normalized to control (mean of femur length in control= 1). (F) ratio of tendon length versus femur total length. No statistical value is available for sr-gal4>UAS-Lifeact.GFP, UAS-vermRNAi flies, as only rare escapers survive until adult stage. Control: $n = 30$ legs (from 10 flies), serp-RNAi: n=42 legs (12 flies), lolal-RNAi: n=20 legs (5 flies). Error bars represent s.d.; $*p < 0.05$ and $***p < 0.001$ (Wilcoxon-Mann-Whitney test). Thus reduction of *serp* expression in tendon cells leads to significantly more elongated tidt and lt than in the control, with a tidt extending up to the most proximal part of the femur. The same observation was made with *verm* RNAi, the three tendons in the femur are longer than WT tendons, with tidt extending to the most proximal part of the femur. Lastly, knockdown of lolal in tendon cells leads to significantly longer tendons compared to WT tendons, extending in the proximal part of femur. Scale bar 50 µm.

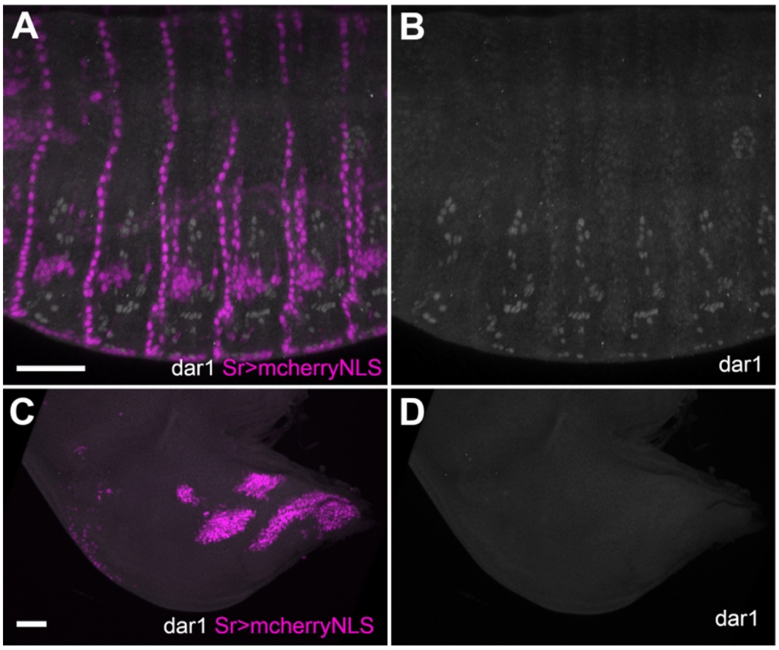

Supplemental Fig. 2. ***dar1*** **expression pattern in** ***Drosophila*** **embryo and wing disc.**
(A-B) Thoracic and abdominal segments of sr-gal4>UAS-mcherryNLS embryo (stage 16) immunostained with anti-mcherry (magenta) and anti-Dar1 (gray) antibodies. *dar1* is expressed in different cell types including glial cells (Ye et al. 2011) but not in muscle attachment sites (magenta). (C-D) Notum region of wing imaginal discs from sr-gal4>UAS-mcherryNLS L3 larvae immunostained with anti-Dar1 antibodies. dar 1 expression (gray) is not expressed in flight muscle tendon precursors (magenta). Scale bar 40 μm.

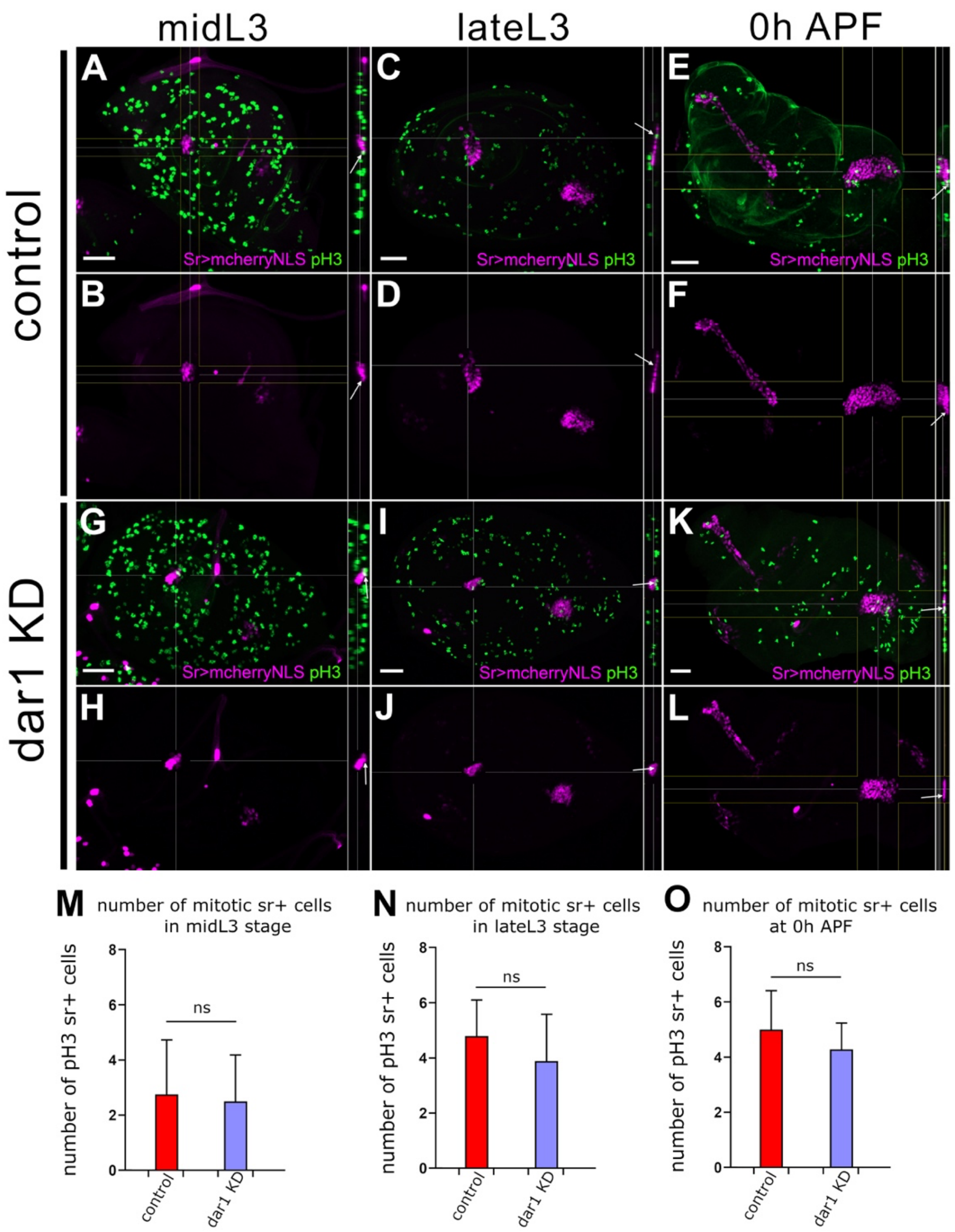

Supplemental Fig. 3. *dar1* knockdown in tendon precursor cells does not alter the number of mitotic cells.

Optical sections of sr-gal4,dar1$^{3010/+}$>UAS-mcherryNLS and sr-gal4,dar1$^{3010/+}$>UAS-mcherryNLS,UAS-dar1RNAi leg discs immunostained with anti-pH3 (green) at different times of development, and lateral views focused on the long tendon of tarsi or tilt in dorsal femur. (A–F) In control leg discs, at midL3 stage (A, B) numerous mitotic cells can be detected, but only a few of them correspond to tendon precursors (arrow). From late L3 stage (C, D) to 0h APF (E, F), the total number of mitotic events decreases and one or two proliferating tendon precursors are occasionally found (arrow). In *dar1* KD leg disc, at midL3, we also observed rare mitotic tendon cells (arrow). At late L3 stage and 0h APF (I-L), the number of tendon cells is strongly reduced compared to the control, but we can still occasionally detect a few mitotic tendon cells (arrow) as in control leg discs. (M-O) Graphs showing the mean number of sr>mcherryNLS cells that are pH3 positive per disc at different stages of development in control and *dar1* KD leg discs. Error bars represent s.d. (student t-test). Observed discs: control *n*=22, *dar1* KD, *n*=24. Scale bar 30 µm.

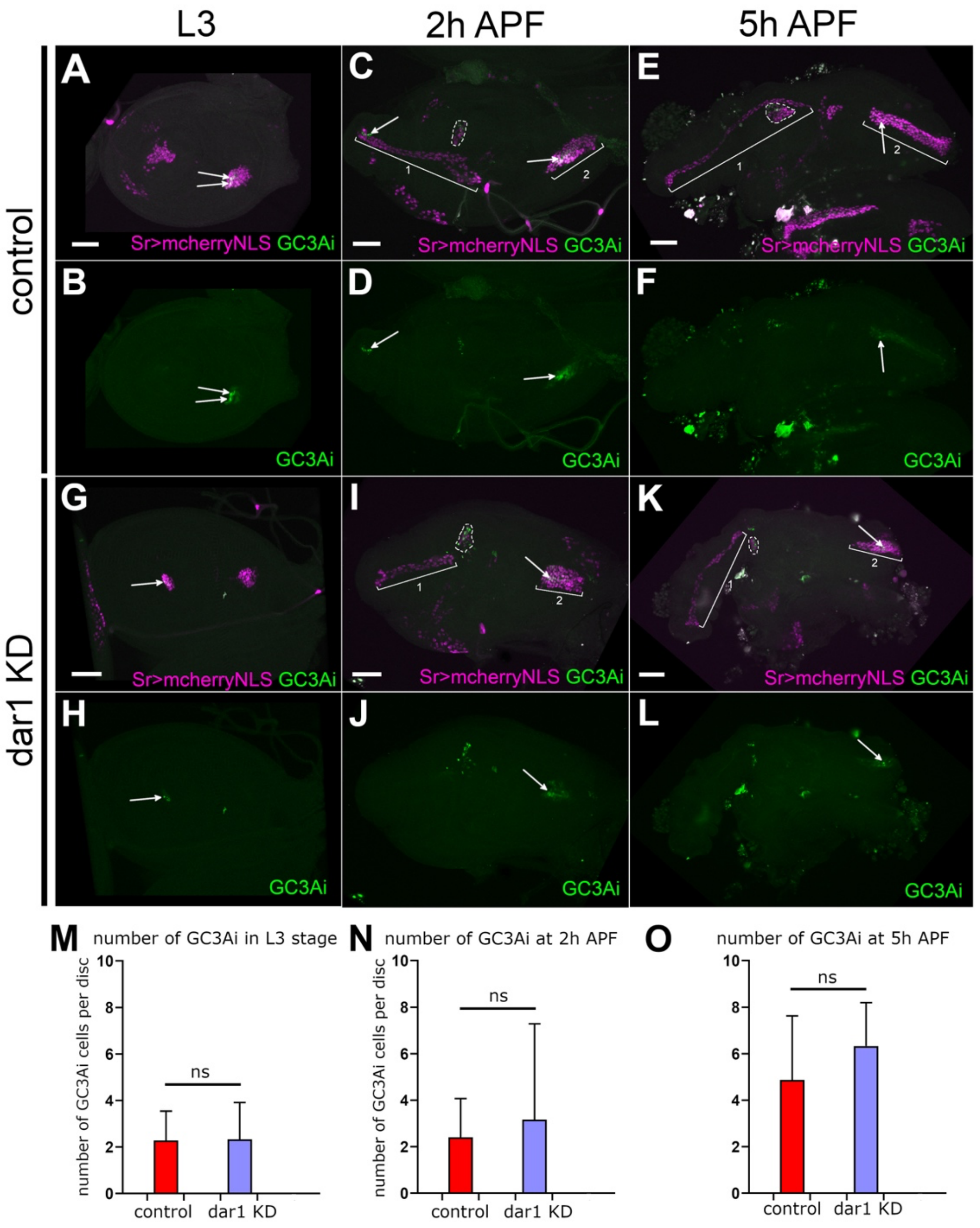

Supplemental Fig. 4. reduction of tendon cells number is not due to ectopic apoptosis. Optical sections of leg discs from progenies of sr-gal4,dar1$^{3010/+}$>UAS-mcherryNLS flies crossed with UAS-GC3Ai (A-F) or UAS-GC3Ai ; UAS-dar1RNAi (G-L). GC3Ai encodes for GFP sensor responding to caspase-3-like protease activity. When expressed in tendon precursors (magenta) using sr-gal4 driver, apoptotic pathway induction is revealed by GFP fluorescence. At third larval instar, in both control (A, B) and *dar1* KD leg disc (G, H), green fluorescence can be observed in a few tendon cells (arrows). During metamorphosis, at 2h APF and then 5h APF, long tendon of the tarsi (bracket 1) and tilt in dorsal femur (bracket 2) appear shorter in *dar1* KD leg discs (I, K) than in the control (C, E) with fewer tendon cells. In both contexts, a few apoptotic cells are observed. Of note, in other newly-specified tendons such those in the tibia (dashed lines), some apoptotic bodies are also observed, with no apparent difference between *dar1* KD and the control context. (M-O) Graphs showing the mean number of GC3Ai positive cells per disc at different stages of development in control and *dar1* KD leg discs. Error bars represent s.d. (student t-test). Observed discs: control *n*=20, *dar1* KD, *n*=21. Scale bar 50 μm.

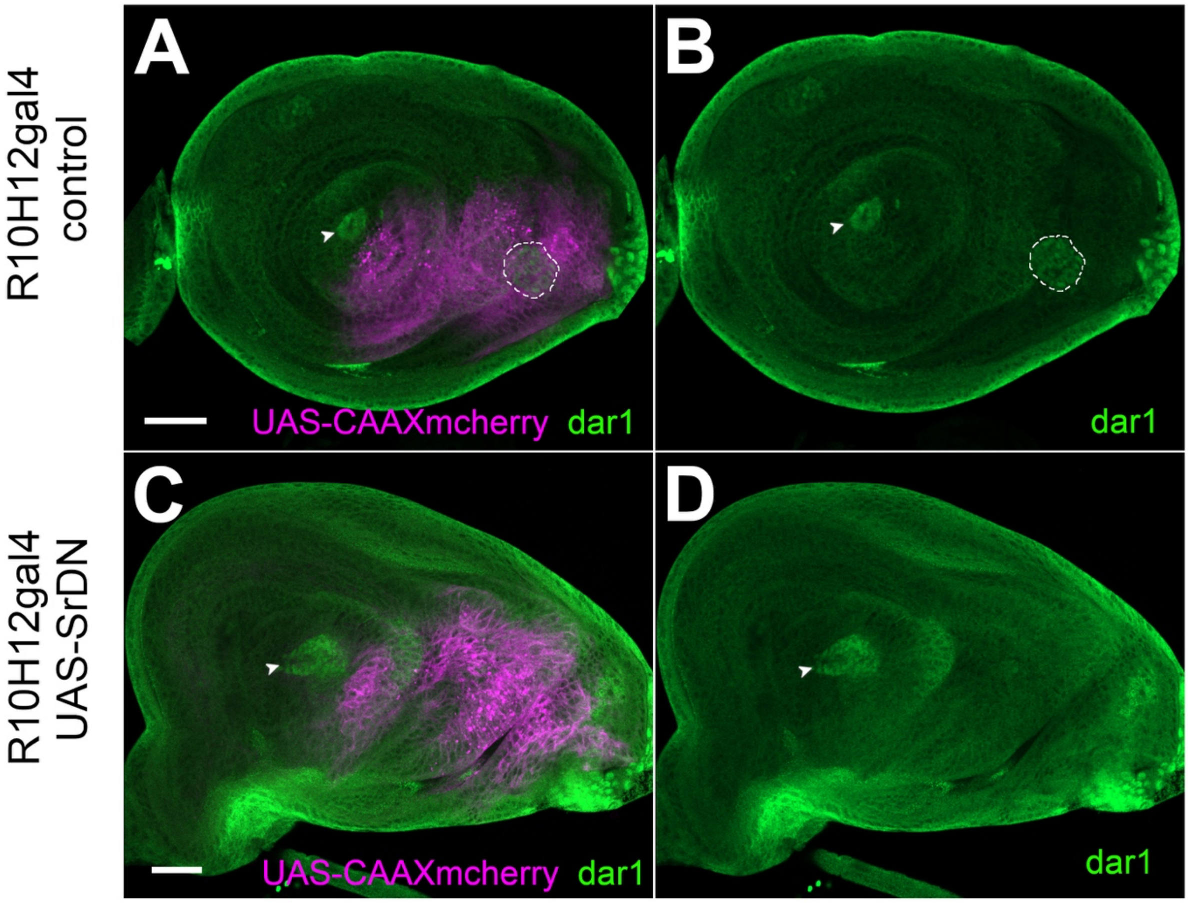

Supplemental Fig. 5. Ectopic expression of a stripe dominant negative form abolishes *dar1* expression.

Confocal sections of R10H12-gal4>UAS-mcherryCAAX leg disc (A-B) and R10H12-gal4>UAS-mcherryCAAX, UAS-SrDN leg disc (C-D) immunostained with anti-Dar1 (green) at third larval instar. UAS-CAAXmcherry expression (magenta) reveals the pattern of R10H12-gal4 driver in dorsal epithelium leg disc overlapping with tendon precursors of the tilt (Laddada et al. 2019) (dashed lines in A and B). When UAS-SrDn is expressed using R10H12gal4 driver, *dar1* expression is completely abolished in the dorsal femur (C-D), whereas *dar1* expression is still detected in the long tendon of tarsi and even occasionally in a wider area (arrowhead in A, B, C and D) suggesting that dominant negative effect of StripeDN expression in surrounding cells might cell non-autonomously affect the patterning of this tendon. Observed discs: control *n*=14, *dar1* KD, *n*=10. Scale bar 30 µm.
